# Hsc70-4: An unanticipated mediator of dsRNA internalization in *Drosophila*

**DOI:** 10.1126/sciadv.adv1286

**Published:** 2025-05-16

**Authors:** Sabrina J. Fletcher, Eugenia S. Bardossy, Lorena Tomé-Poderti, Thomas Moss, Vanesa Mongelli, Lionel Frangeul, Hervé Blanc, Yann Verdier, Joelle Vinh, Shaeri Mukherjee, Maria-Carla Saleh

**Affiliations:** ^1^ Viruses and RNAi Unit, Institut Pasteur, Université Paris Cité, CNRS UMR3569, F-75015 Paris, France.; ^2^ Department of Microbiology and Immunology, The George William Hooper Foundation, University of California, San Francisco, San Francisco, CA 94143, USA.; ^3^ Biological Mass Spectrometry and Proteomics (SMBP), ESPCI Paris, PSL University, CNRS, 75005 Paris, France.; ^4^ Chan Zuckerberg Biohub, San Francisco, CA 94158, USA.

## Abstract

The small interfering RNA pathway is the primary antiviral defense mechanism in invertebrates and plants. This systemic mechanism relies on the recognition, transport, and internalization of double-stranded RNA (dsRNA). Our aim was to identify cell surface proteins that bind extracellular dsRNA and mediate its internalization in *Drosophila* cells. We used coimmunoprecipitation coupled with proteomics analysis and found that silencing heat shock cognate protein 70-4 (Hsc70-4), a constitutively expressed heat shock protein, impairs dsRNA internalization. Unexpectedly, despite lacking a predicted transmembrane domain, Hsc70-4 localizes to the cell membrane via lipid interactions. Antibody blocking experiments revealed an extracellular domain on Hsc70-4 that is essential for dsRNA internalization. Intriguingly, this dsRNA-specific binding capacity of Hsc70-4 functions independently of its chaperone activity. These findings not only highlight Hsc70-4 as a previously uncharacterized and essential component in the dsRNA internalization process but also offer promising insights for advancing RNA interference–based technologies to combat pests and vector-borne diseases.

## Teaser

Hsc70-4 localizes to the cell surface of *Drosophila* cells, binds to exogenous dsRNA, and mediates its internalization.

## INTRODUCTION

Systemic immunity is a defense mechanism that is triggered to fight pathogenic infections while contributing to resistance to infection in noninfected cells or tissues (*1*–*3*). This type of immune response is critical to maintain homeostasis. One of the main characteristics of systemic immunity is the transmission of a signal from infected to noninfected cells/tissues to trigger a broader immune response (*2*, *3*). During their replication cycles, viruses produce double-stranded RNA (dsRNA) intermediates (*4*). In insects, plants, and nematodes, sensing and cleavage of virus-derived dsRNA into small interfering RNAs (siRNAs) by intracellular proteins trigger an RNA interference (RNAi)–mediated immune response that controls viral replication through the siRNA pathway.

The antiviral siRNA pathway has been most extensively studied in the model insect, *Drosophila melanogaster* (*5*–*8*). Specifically, in vivo experiments in adult flies revealed that virus-derived dsRNA is transmitted from infected to noninfected cells, where it is processed into siRNAs and confers protection against *Drosophila* C virus (DCV) or Sindbis virus infection (*9*, *10*).

Even though intracellular RNAi processes are well understood, the mechanisms of release, transmission, sensing, and internalization of dsRNA are unclear. While the release of dsRNA from infected cells is thought to be a consequence of cell lysis caused by viral infection (*9*), how dsRNA is internalized by noninfected cells remains obscure. Hemocytes isolated from both larvae and adult flies can internalize naked extracellular dsRNA, causing activation of the siRNA pathway (*10*, *11*). Accordingly, in vitro experiments using the hemocyte-like *D. melanogaster* Schneider 2 (S2) cell line revealed that these cells can also internalize dsRNA by receptor-mediated endocytosis under certain conditions (*12*, *13*). These findings imply the existence of a receptor that binds naked extracellular dsRNA at the cell surface before internalization. Several proteins have already been proposed as dsRNA receptors in other organisms, such as the systemic RNAi-defective-2 protein (SID-2) in *Caenorhabditis elegans* (*14*), the scavenger receptor class A (SR-A), and macrophage-1 antigen (Mac-1) in humans (*15*–*18*). In insects, scavenger receptors have also been found to mediate the early steps in the internalization of dsRNA (*12*, *19*). Specifically, in *D. melanogaster*, two scavenger receptors, SR-CI and Eater (*13*), have been proposed to play roles in this process. However, experimental data showed that these receptors are mainly involved in general phagocytosis and not in the specific internalization of dsRNA by endocytosis (*20*).

Here, we aimed to identify the cell surface protein(s) responsible for binding extracellular dsRNA before its internalization in *D. melanogaster*. To this end, we developed an in vitro model using the *Drosophila* S2 cell line. We used two different proteomic approaches to identify candidate proteins. One approach was designed to identify total cell surface proteins, while the other approach was designed to specifically pull down dsRNA-protein complexes at the cell surface. Through a functional screen of candidate proteins, we identified heat shock cognate protein 70-4 (Hsc70-4) as a protein that localizes to the cell surface and can bind dsRNA. Hsc70-4 is part of the pleiotropic heat shock protein family (*21*). The heat shock cognate proteins differ from the more well-known heat shock proteins in that they are constitutively expressed (*22*, *23*). Although this protein family has been studied extensively and is thought to function primarily as a chaperone, unexpected roles for heat shock cognate proteins are still being identified (*24*–*26*). Our findings demonstrate that Hsc70-4 plays a previously uncharacterized role in the first steps of dsRNA internalization at the cell surface of S2 cells.

## RESULTS

### Identification of dsRNA binding cell surface proteins in S2 cells

**Fig. 1.**
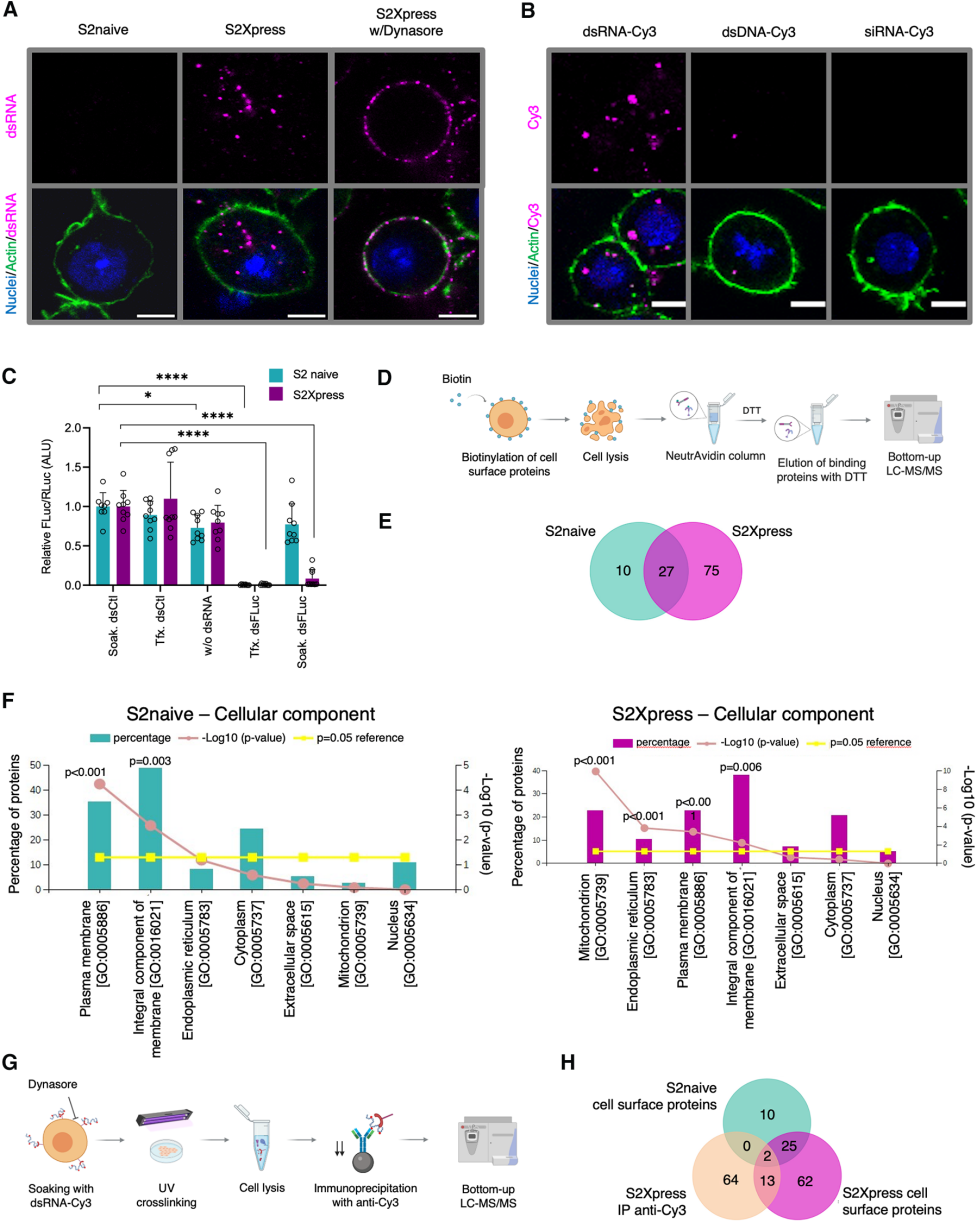
S2 cell model and proteomic approaches to identify cell surface dsRNA binding proteins. (**A**) Internalization of dsRNA-Cy3 evaluated by fluorescence confocal imaging of single Z-sections. dsRNA soaking was done for 40 min. Actin is in green, and nuclei are in blue. Dynasore was added for 1 hour before dsRNA soaking. (**B**) Specificity of nucleic acid internalization by S2Xpress cells. dsRNA-Cy3 (dsFLuc), dsDNA-Cy3 (FLuc), or siRNA-Cy3 (siGAPDH) (magenta) was added during soaking. Staining is as in (A). (**C**) Silencing of firefly luciferase by internalized dsRNA. Cells were cotransfected with plasmids expressing firefly and *Renilla* luciferase followed by soaking with dsRNA targeting firefly luciferase (dsFLuc) or GFP (dsCtl). As positive controls, dsRNAs and plasmids were cotransfected. Luciferase activity was measured 24 hours after induction. Firefly luciferase values were normalized to *Renilla* luciferase values. The histogram shows the mean + SD firefly/*Renilla* ratio relative to soaking with dsCtl (Soak. dsCtl) from three independent experiments (*n* = 8 to 9). Welch’s ANOVA was used to detect significant differences compared to Soak. dsCtl. (**D**) Protocol used to purify cell surface proteins from S2naive and S2Xpress cells. (**E**) Venn diagram showing proteins identified from S2naive and S2Xpress cells in (D). (**F**) Cellular component analysis of the proteins identified by the protocol in (D). Statistical analysis was done with FunRich (*57*) (hypergeometric test). (**G**) Protocol used to purify dsRNA binding proteins from S2Xpress cells by IP. Dynasore was used to prevent internalization of dsRNA-Cy3. (**H**) Venn diagram showing proteins identified in S2naive and S2express cells using the different proteomic protocols. Venn diagrams were done with FunRich (*57*). Protocol schemes were created with BioRender.com. Scale bars represent 5 μm. ALU, arbitrary luciferase units. *P < 0.05; **P < 0.01; ****P < 0.0001.

*D. melanogaster* S2 cells are widely used as a tool to study gene function because of their capacity to internalize dsRNA from the cell medium to induce gene silencing through RNAi. dsRNA internalization in S2 cells depends on the composition of the growth medium and on the specific cell line used. While S2 cells internalize dsRNA from the growth medium only in the absence of fetal bovine serum (FBS), the S2 receptor plus (S2R+) cells (*27*) can internalize dsRNA in the presence or absence of FBS (*28*). S2R+ cells are persistently infected with DCV, DAV (*Drosophila* A virus), DXV (*Drosophila* X virus), and FHV (Flock House virus) (*29*). To avoid the confounding effects of virus infection, we decided to develop a model for dsRNA internalization in noninfected S2 cells (hereafter referred to as S2naive), as determined by deep sequencing. Considering that FBS prevents dsRNA internalization by S2 cells, we adapted our S2naive cell line to growth in Insect-XPRESS protein-free medium, which does not contain FBS. We then tested dsRNA internalization in the newly adapted cells (hereafter referred to as S2Xpress), which show similar growth, viability, and apoptosis rate to S2naive cells (fig. S1, A and B). First, we visualized internalization of dsRNA labeled with Cy3 (dsRNA-Cy3) using fluorescence confocal imaging. Cells were soaked with dsRNA-Cy3, followed by extensive washing. dsRNA-Cy3 labeling was confirmed by an electrophoretic shift of the labeled dsRNA compared to unlabeled dsRNA on an agarose gel and by immunofluorescence with the anti-dsRNA antibody, J2 (fig. S1, C and D). We found that S2Xpress cells, but not S2naive cells, internalized dsRNA-Cy3 (Fig. 1A). Receptor-mediated endocytosis was previously suggested to be involved in dsRNA internalization in S2 cells (*12*, *13*). To determine whether this was also the case for S2Xpress cells, we tested the effect of Dynasore, an inhibitor of dynamin-dependent endocytosis, on the internalization of dsRNA. We treated cells with Dynasore before addition of dsRNA-Cy3 to the culture medium. While dsRNA-Cy3 was detected as a punctuated pattern within the cytoplasm of S2Xpress cells, dsRNA-Cy3 was localized to the cell surface when cells were treated with Dynasore (Fig. 1A), suggesting that dynamin-dependent endocytosis is essential for uptake. Furthermore, dsRNA internalization by S2Xpress cells was not sequence dependent because dsRNA internalization was still evident when a dsRNA harboring a different sequence was used (fig. S1E). Furthermore, we found that internalization of nucleic acids by S2Xpress cells was restricted to dsRNA, because very few or no spots were seen inside cells when we performed soaking with dsDNA-Cy3 or siRNA-Cy3 (Fig. 1B).

We then assessed the ability of the internalized dsRNA to trigger RNAi-based silencing in a luciferase/*Renilla* assay. Cells expressing firefly and *Renilla* luciferases were soaked with dsRNA targeting the firefly luciferase sequence (dsFluc) or an unrelated control sequence [dsCtl, with a green fluorescent protein (GFP) sequence]. Firefly luciferase activity was quantified as an indirect measure of dsRNA internalization, and the *Renilla* luciferase level was used for normalization. We found a significant decrease in firefly luciferase activity for S2Xpress cells treated with dsFluc compared to dsCtl (Fig. 1C). As expected, dsFluc treatment did not significantly alter firefly luciferase activity in S2naive cells grown in the presence of FBS (Fig. 1C). Furthermore, dsRNA internalization was dose dependent in S2Xpress cells (fig. S1F). We also found that substituting Insect-XPRESS protein-free medium for 10% FBS-Schneider medium during 4-hour incubation with dsRNA in S2Xpress cells had little effect on the internalization, as significant silencing was still observed under these conditions (fig. S1G). These results indicate that FBS does not interfere with binding and internalization of dsRNA in S2Xpress cells. As expected, the presence of FBS had a much greater impact on dsRNA internalization for S2naive cells (fig. S1G). We note that while some silencing was observed for S2naive cells in the absence of FBS, silencing levels were minute compared to those observed in S2Xpress cells.

Together, these results show that S2Xpress cells can specifically internalize dsRNA from their environment to trigger the siRNA pathway. Because S2Xpress cells were obtained after adaptation of S2naive cells to a protein-free medium, and because only S2Xpress cells can internalize dsRNA, comparison of S2naive cells with S2Xpress cells provided us with an appropriate model system to search for proteins involved in dsRNA binding and internalization in *D. melanogaster*.

Two scavenger receptors, SR-CI and Eater, have been previously described to mediate the internalization of dsRNA in S2 cells (*13*). We sought to test whether these proteins affected internalization by knockdown of Eater and SR-CI followed by luciferase-based assay. We were not able to produce dsRNA corresponding to SR-CI because we could not obtain a reverse transcription polymerase chain reaction (RT-PCR) product from S2Xpress RNA (fig. S1H). This precluded us from performing a dsRNA-based silencing assay for SR-CI. For Eater, we tested dsRNA internalization under knockdown conditions by high-content fluorescence quantification and confocal imaging, but we did not see an effect on dsRNA internalization when we silenced this protein in S2xpress cells (fig. S1, H to J). Therefore, we could not confirm the roles of SR-CI or Eater in naked dsRNA uptake in S2Xpress cells.

To evaluate differences in cell surface components between S2naive and S2Xpress cells, we purified the cell surface proteins from both cell lines and determined their identity by liquid chromatography–tandem mass spectrometry (LC-MS/MS) (Fig. 1D). We were able to identify 37 proteins in extracts from S2naive cells and 102 proteins in extracts from S2Xpress cells (Fig. 1E and table S1). Only 27 of these proteins were present in both cell types. A cellular component analysis showed a high percentage of known plasma membrane proteins detected for both cell types, validating the purification protocol (Fig. 1F). For S2Xpress cells, a high percentage of mitochondrial proteins was also detected. We also found some cytoplasmic and nuclear proteins in both cell types, which we speculate could be due to the presence of dead cells or coelution of binding partners at the plasma membrane.

To specifically purify the dsRNA binding proteins at the cell surface, we developed a state-of-the-art immunoprecipitation (IP) protocol based on the CLIP assay (cross-linking and IP) (Fig. 1G). We used Dynasore to inhibit the internalization of dsRNA-Cy3 by S2Xpress cells, thereby sequestering a high concentration of dsRNA-Cy3 at the cell surface. We showed that dsRNA-Cy3 is functional in luciferase assays, confirming that Cy3 labeling does not alter the dsRNA internalization mechanism and processing (fig. S2A), as previously demonstrated (*12*). Next, we ultraviolet (UV) cross-linked dsRNA-Cy3 to interacting proteins and performed IP using an anti-Cy3 antibody that we found to have high specificity for dsRNA-Cy3 (fig. S2B). LC-MS/MS analysis of purified proteins allowed us to identify 79 proteins (Fig. 1H and table S2). When we compared the identified proteins with those found in the cell surface pull-down assay, we found that 13 of them were also found on the surface of S2Xpress cells, and only 2 were found on the surfaces of both cell types (S2naive and S2Xpress). Cellular component analysis showed that several of the identified proteins corresponded to mitochondrial proteins and plasma membrane proteins (fig. S2C) as well as several nuclear proteins. This could be explained by the fact that dsRNA added to the medium bound to some dying cells during incubation (fig. S2D). In addition, coelution of binding partners of dsRNA binding proteins could explain the presence of cytoplasmic proteins, although it is possible that some of them are undescribed plasma membrane proteins. Overall, when examining the molecular functions of the identified proteins, we found a high percentage of RNA binding proteins, indicating that the IP protocol was successful in pulling-down proteins binding to dsRNA.

Together, these two proteomic approaches revealed that there are differences in the compositions of the cell surfaces of S2naive and S2Xpress cells and provided us a list of potential cell surface dsRNA binding proteins. Of note, SR-CI and Eater were not immunoprecipitated by either of the two approaches.

### A functional screen of dsRNA binding protein candidates identified Hsc70-4 as a protein involved in dsRNA internalization

**Fig. 2.**
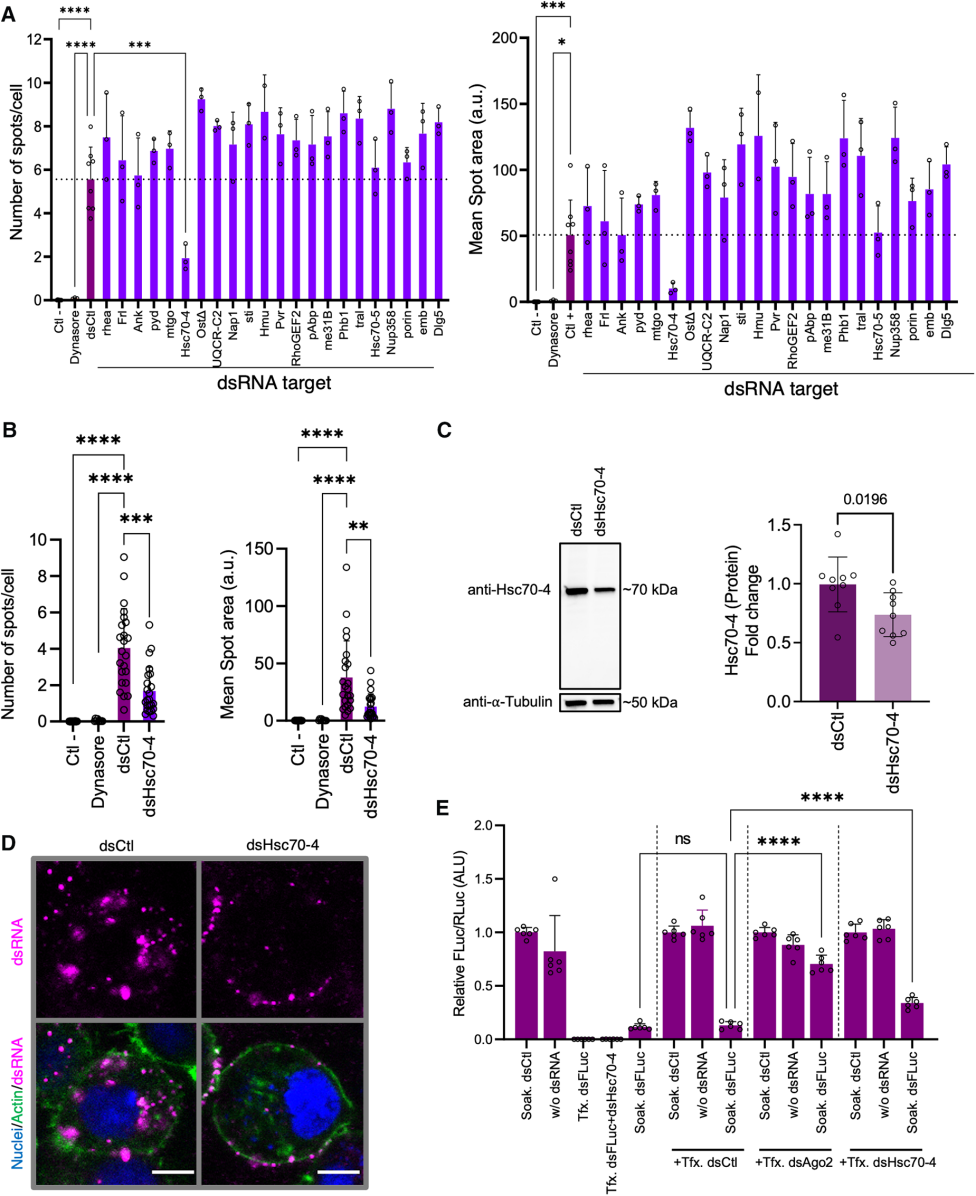
High-content screen of selected candidates. (**A**) Candidate genes were silenced in S2Xpress cells by transfection with gene-specific dsRNA (dsRNA target) or nonspecific dsRNA (Ctl−, dsCtl, Dynasore) followed by soaking with nonspecific dsRNA-Cy3 (dsFLuc) and analyzed with the Opera Phenix High-Content microscope. Cy3 intensity, number of Cy3 spots per cell, and mean spot area were quantified on single Z-sections with Columbus software. Histograms show the means + SD of spots per cell and mean spot area. ANOVA with Dunnett’s post hoc tests was used to detect significant differences compared to dsCtl (Ctl− and dsCtl, *n* = 8; dsCandidates and Dynasore, *n* = 3; dsHmu, *n* = 2). *P* values are indicated in fig. S2B. a.u., arbitrary units. (**B**) High-content analysis to determine the effects of silencing Hsc70-4 on dsRNA internalization. Experiments and analyses were performed as in (A). Data are from three independent experiments (Ctl−, *n* = 24; Dynasore, *n* = 9; dsCtl, *n* = 23; dsHsc70-4, *n* = 23) (Welch’s ANOVAs with Dunnett’s T3 post hoc). (**C**) Immunoblot and quantification of anti-Hsc70-4 signal in S2Xpress cells transfected with dsRNA targeting Hsc70-4 (dsHsc70-4) compared to the control (dsCtl). Tubulin was used as a control. Histograms show the means + SD normalized to tubulin (unpaired *t* test, *n* = 9). (**D**) Confocal imaging of single Z-sections to confirm the findings shown in (B). S2Xpress cells were transfected with dsHsc70-4 or dsCtl for 72 hours followed by soaking of dsRNA-Cy3 (magenta). Actin is in green, and nuclei are in blue. Scale bars represent 5 μM. (**E**) Effect of down-regulation of Hsc70-4 on silencing by luciferase assay. Cells were transfected with dsHsc70-4, dsCtl, or dsAgo2 (as a positive control of effect on silencing). Experiments were done as in Fig. 1C. The histogram shows the mean + SD firefly/*Renilla* ratio relative to Soak. dsCtl from two independent experiments (*n* = 6). ANOVA with Dunnett’s post hoc tests was used to detect significant differences compared to dsCtl. *P < 0.05; **P < 0.01; ***P < 0.001; ****P < 0.0001. ns, not significant.

We next performed an in silico analysis of the proteins identified by our two proteomic approaches to select candidates to test in S2Xpress cells. As a selection strategy, we considered protein localization, type of protein, and presence in the results of both proteomic strategies. Through this process, we produced a list of 22 candidate proteins to test for their possible roles in mediating dsRNA internalization. To this end, we performed a high-content imaging screen based on silencing candidate proteins by transfection with candidate gene–specific dsRNA followed by incubation with nonspecific dsRNA-Cy3. The Cy3 signal inside cells was evaluated. We quantified total Cy3 intensity, the number of Cy3 spots inside the cytoplasm, and the size of the spots as an indicator of dsRNA-Cy3 internalization (Fig. 2A and fig. S3A). We successfully identified one candidate, Hsc70-4 (CG4264), that impaired the internalization of dsRNA when silenced (Fig. 2, A and B, and fig. S3, A to C). We confirmed silencing of Hsc70-4 by RT-PCR (fig. S3D) and by Western blot (Fig. 2C) using a polyclonal antibody developed against *Drosophila* Hsc70-4. The specificity of the anti-Hsc70-4 antibody was verified by IP followed by protein identification using LC-MS/MS (fig. S3E and table S3). Furthermore, confocal imaging analysis confirmed that when Hsc70-4 was silenced, there was a decrease in the internalization of dsRNA-Cy3 (Fig. 2D). In addition, we found that down-regulating Hsc70-4 in S2Xpress cells significantly decreased luciferase silencing (Fig. 2E). Our proteomics assays identified Hsc70-4 as both a dsRNA binding protein and a cell surface protein in S2Xpress cells but not in S2naive cells. To confirm the presence of Hsc70-4 as a membrane protein of S2 cells, we performed a proteomic analysis of total membrane extracts of S2naive and S2Xpress cells using LC-MS/MS (fig. S4A and table S4). As expected, we were able to detect Hsc70-4 in both cell extracts, confirming that Hsc70-4 is a membrane protein. To better understand the differences between S2naive and S2Xpress cells, we compared the expression levels of Hsc70-4 by RT-qPCR (reverse transcription quantitative polymerase chain reaction). We found that S2naive cells had significantly greater expression of Hsc70-4 than S2Xpress cells (fig. S4B). In Western blot, we found that S2naive and S2Xpress cells present similar total levels of Hsc70-4 protein (fig. S4C), suggesting that the difference at the transcript level could be due to different posttranscriptional regulation mechanisms. Overall, these results highlight Hsc70-4 as a key factor in dsRNA internalization in S2Xpress cells and suggest that the difference between S2naive cells and S2Xpress cells regarding Hsc70-4 could relate to its localization, binding partners, or posttranslational modifications.

### Hsc70-4 localizes to the cell surface of S2 cells and binds specifically dsRNA in vitro

**Fig. 3.**
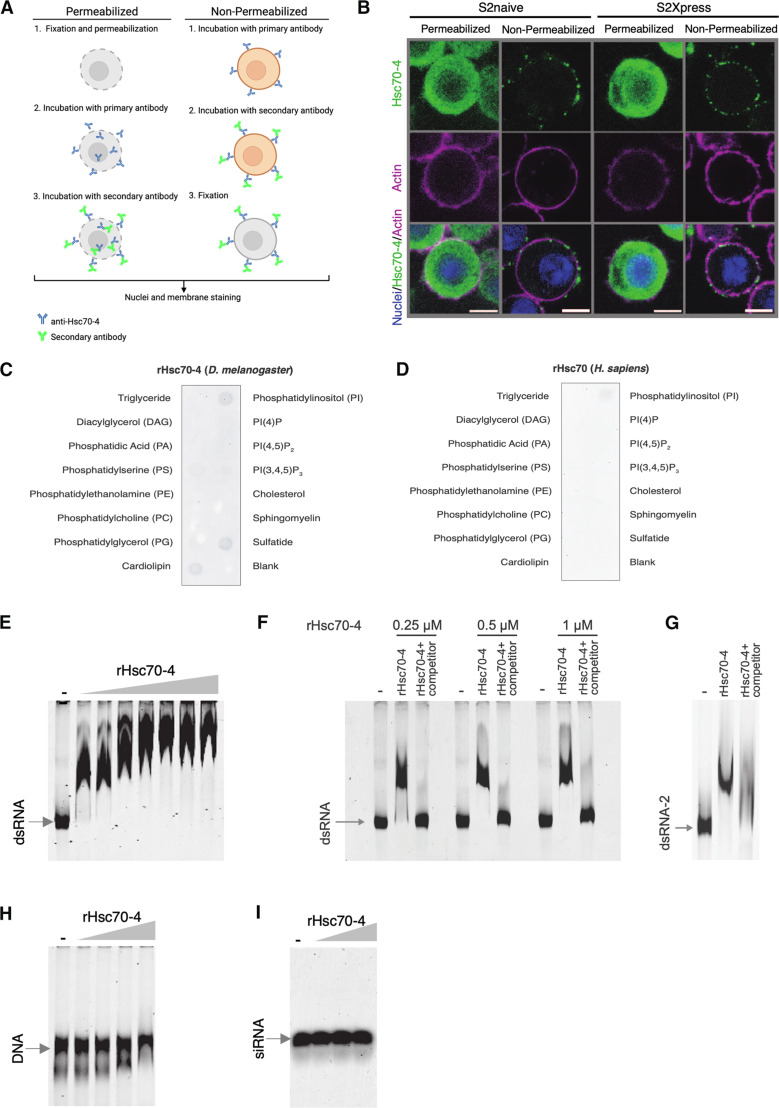
Cell surface localization of Hsc70-4 and binding to dsRNA. (**A**) Schematic representation of immunofluorescence staining performed with anti-Hsc70-4 on S2 cells under permeabilized and nonpermeabilized conditions to detect Hsc70-4 localization. The cartoon was created using BioRender.com. (**B**) Fluorescence confocal images of single Z-sections of S2naive and S2Xpress under permeabilized and nonpermeabilized conditions. Hsc70-4 is in green, actin is in magenta, and nuclei are in blue. (**C** and **D**) PIP Strip membranes were used to detect the binding of *Drosophila* recombinant protein (rHsc70-4) or its human ortholog (rHsc70) to various lipids. (**E**) EMSA assay testing binding of rHsc70-4 (0.25, 0.5, 1, 2, 3, 4, and 5 μM) to dsRNA-Cy3 (dsCat, 0.76 nM). The electrophoretic shift of dsRNA-Cy3 was evaluated by native PAGE. A mobility shift of dsRNA-Cy3 incubated with rHsc70-4 confirms binding. (**F**) In a competition assay, unlabeled dsRNA and Cy3-labeled dsRNA were incubated with rHsc70-4 (0.25, 0.5, and 1 μM) in a 10:1 unlabeled:labeled dsRNA ratio. An increase in the mobility of Cy3-labeled dsRNA confirmed that unlabeled dsRNA displaced the labeled dsRNA. (**G**) EMSA using a dsRNA with a different sequence (dsRNA-2). (**H**) EMSA of dsDNA-Cy3 (Cat, 0.76 nM) with rHsc70-4 (0.25, 0.5, 1, and 2 μM) as in (E). (**I**) EMSA of siRNA-Cy3 (siGAPDH, 0.76 nM) with rHsc70-4 (0.5, 1, and 2 μM) as in (E). All EMSA experiments were performed at least twice, giving similar results. The first lane in all gels corresponds to Cy3-labeled nucleic acid (dsRNA, dsDNA, or siRNA) incubated without rHsc70-4. Scale bars represent 5 μm.

Hsc70-4 is a member of the heat shock protein 70 (Hsp70) family that is constitutively expressed in *D. melanogaster* (*30*). Hsc70-4 is involved in protein folding and contributes to several processes including clathrin-mediated endocytosis (*31*–*33*) and neurotransmitter exocytosis (*34*). GWAS has linked it to the RNAi response (*35*), although further details are lacking. Hsc70-4 is localized in the cytosol, mitochondria, and nucleus (*36*, *37*). Because our candidate was obtained from a screen of cell surface proteins, we wanted to confirm the subcellular localization of Hsc70-4 in S2 cells. To this end, we performed immunofluorescence staining of S2naive and S2Xpress cells under permeabilized and nonpermeabilized conditions using a specific anti-Hsc70-4 antibody. Under nonpermeabilized conditions, antibodies cannot cross the plasma membrane, facilitating determination of whether the target protein is present at the cell surface (Fig. 3A). As a control for detection of a cell surface protein, we performed immunofluorescence staining of the transmembrane protein H19 in an H19-expressing cell line (designated S2-H19) (fig. S5A). Hsc70-4 showed cell surface localization with a punctate pattern that was similar in both S2Xpress and S2naive cells (Fig. 3B). As expected, under permeabilized conditions, Hsc70-4 localized to the cytoplasm in both cell types (Fig. 3B). These results confirm that Hsc70-4 has both cytoplasmic and cell surface localization. Moreover, overexpression of Hsc70-4 in S2naive and S2Xpress cells by transfection with a plasmid expressing recombinant Hsc70-4 (rHsc70-4) shows that Hsc70-4 accumulates in the cell membrane (fig. S5B). Of note, overexpression of rHsc70-4 did not alter dsRNA internalization capacity in either cell line. Because Hsc70-4 does not have a predicted transmembrane domain, we tested the ability of Hsc70-4 to interact with membrane lipids. To this end, membrane strips were incubated with rHsc70-4. The recombinant human ortholog (rHsc70) was also tested for comparison. Purified *Drosophila* rHsc70-4 was able to bind specifically to cardiolipin, sulfatide, and phosphatidylserine, contrasting with the human ortholog that did not bind these membrane lipids (Fig. 3, C and D). Of note, binding of Hsc70-4 to phosphatidylinositol cannot be interpreted because it was also seen in the antibody controls (fig. S5C). Overall, our results suggest that Hsc70-4 behaves as a peripheral membrane protein that anchors to the cell membrane by interacting with the lipid bilayer.

Several different domains have been identified as dsRNA binding domains, including the αβββα fold commonly known as the dsRNA binding domain, the helicase domain, and the nucleotidyltransferase domain (*38*, *39*). Hsc70-4 does not present a previously identified dsRNA binding domain. Nevertheless, we tested whether rHsc70-4 can bind dsRNA in vitro. Different concentrations of purified rHsc70-4 were incubated with dsRNA-Cy3, and binding was assessed by electrophoretic mobility shift assays (EMSAs). We found that rHsc70-4 binds dsRNA in vitro, with increasing concentrations of rHsc70-4 resulting in decreased mobility of dsRNA-Cy3 compared to dsRNA-Cy3 alone (Fig. 3E). Moreover, the fact that the electrophoretic shift gradually increased with increasing concentrations of rHsc70-4 until reaching a full shift may indicate that more than one molecule of rHsc70-4 can bind to each molecule of dsRNA-Cy3 or that Hsc70-4 oligomerizes on the dsRNAs. A competitor assay with unlabeled dsRNA was performed to confirm binding specificity. Here, we observed increased mobility for dsRNA-Cy3 in the presence of competitor dsRNA compared to the assay without competitor dsRNA, indicating that unlabeled dsRNA can displace labeled dsRNA (Fig. 3F). By using a dsRNA with a different sequence as a probe, we confirmed that binding of dsRNA to rHsc70-4 was not sequence dependent (Fig. 3G). Moreover, the electrophoretic mobility of dsDNA-Cy3 or siRNA-Cy3 was not altered by incubation with rHsc70-4 (Fig. 3, H and I). When we tested dsRNAs of different lengths, we observed that 50– and 100–base pair (bp) dsRNAs did not bind Hsc70-4, whereas 250- and 500-bp dsRNAs did (fig. S5D). These results confirm that rHsc70-4 can bind dsRNA in vitro and that this binding is specific to dsRNA longer than 100 bp.

### Chaperone activity does not mediate the role of Hsc70-4 in dsRNA binding and internalization

**Fig. 4.**
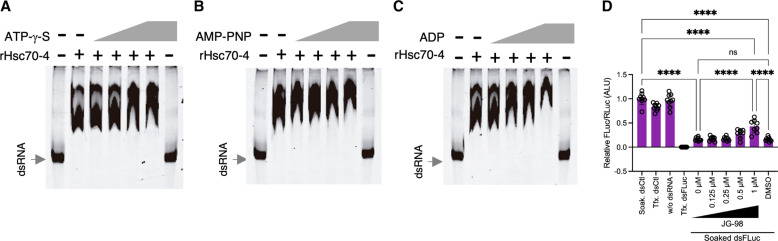
Effect of nucleotide substrates on rHsc70-4 binding to dsRNA and JG-98 treatment on silencing of luciferase. EMSAs testing the binding of rHsc70-4 (2 μM) to dsRNA-Cy3 (dsCat, 0.76 nM) in the presence of (**A**) ATP-γ-S, (**B**) AMP-PNP, or (**C**) ADP. Concentrations of 1, 2.5, 5, and 10 mM of each substrate were used. The electrophoretic shift of dsRNA-Cy3 was assessed by native PAGE. All EMSA experiments were performed at least three times, giving similar results. The first lane in all gels corresponds to dsRNA-Cy3 incubated without rHsc70-4. The last lane in all gels corresponds to dsRNA-Cy3 incubated without rHsc70-4 and with each substrate (10 mM). (**D**) Effect of pretreatment of S2Xpress cells with a JG-98 inhibitor on silencing of luciferase. Experiments were performed as in Fig. 1C. After transfection with luciferase-expressing plasmids, cells were incubated with JG-98 (0.125, 0.25, 0.5, and 1 μM) or DMSO (1 μM). Then, dsFluc or dsCtl was added to the media. Last, luciferase activity was measured. As positive and negative controls of RNAi silencing, dsFluc and dsCtl were cotransfected, respectively. The histogram shows the mean + SD firefly/*Renilla* ratio relative to Soak. dsCtl from three independent experiments (*n* = 9). ANOVA followed by Tukey’s post hoc test was used to detect significant differences between treatments. **P < 0.01; ***P < 0.001; ****P < 0.0001.

Hsp70 proteins share a highly conserved bipartite domain structure composed of an adenosine triphosphatase (ATPase) domain and a substrate-binding domain (*40*). The chaperone activity of Hsp70 is based on the adenosine 5′-triphosphate (ATP)–dependent allosteric conformational change of Hsp70 (*40*). To test whether the role of Hsc70-4 at the cell surface depends on its chaperone activity, we first investigated whether the binding of Hsc70-4 to dsRNA is ATP dependent. We used several nucleotide substrates known to bind to the ATPase domain of Hsp70s and prevent chaperone-associated conformational changes, thereby inhibiting chaperone activity. rHsc70-4 was incubated with dsRNA-Cy3 in the presence of increasing concentrations of (i) adenosine 5′-*O*-(3-thiotriphosphate) (ATP-γ-S), a slowly hydrolyzable ATP analog; (ii) adenylyl-imidodiphosphate (AMP-PNP), a nonhydrolyzable ATP analog; or (iii) adenosine 5′-diphosphate (ADP). After incubation, the binding of rHsc70-4 dsRNA-Cy3 was assessed by EMSA. We observed that ATP-γ-S, AMP-PNP, or ADP did not reduce the mobility of dsRNA-Cy3 at the concentrations tested (Fig. 4, A to C). This result indicates that the ability of rHsc70-4 to bind dsRNA in vitro is ATP independent.

We next investigated whether the role of Hsc70-4 in the internalization of dsRNA could be inhibited pharmacologically. To this end, we used the compound JG-98 in a luciferase/*Renilla* assay. JG-98 is an allosteric inhibitor of Hsp70 that binds tightly to a conserved site on Hsp70 and prevents the conformational change from the ADP to ATP state (*41*). S2Xpress cells were incubated with increasing concentrations of JG-98 and then soaked with dsFluc. We found that pretreatment of S2 cells with JG-98 did not prevent silencing of luciferase compared to the dimethyl sulfoxide (DMSO)–treated control, indicating that dsRNA was able to enter the cells (Fig. 4D). Together, these results show that the role of Hsc70-4 in dsRNA binding and internalization is not mediated by its chaperone activity.

### Blocking Hsc70-4 with anti-Hsc70-4 prevents dsRNA internalization

**Fig. 5.**
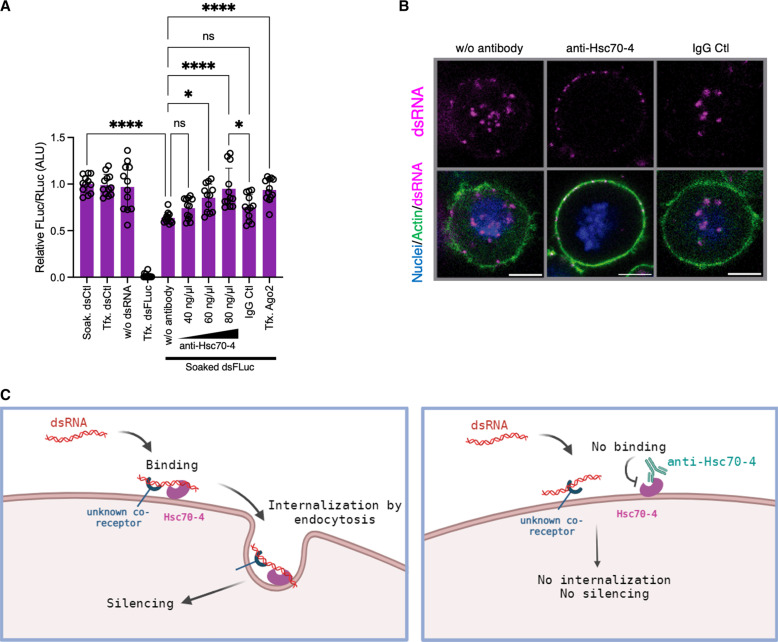
Blocking of dsRNA internalization with an anti-Hsc70-4 antibody. (**A**) Effect of pretreatment of S2Xpress cells with an anti-Hsc70-4 antibody on silencing of luciferase. Experiments were performed as in Fig. 1C. After transfection with luciferase-expressing plasmids, cells were incubated with anti-Hsc70-4 (40, 60, and 80 ng/μl) or an unrelated IgG antibody (80 ng/μl) as a control for 1 hour. Cells were then washed with PBS, and dsFluc or dsCtl was added to the media. Last, luciferase activity was measured. For positive and negative controls of RNAi silencing, dsFluc and dsCtl were cotransfected, respectively. As a positive control of inhibition of RNAi silencing, dsAgo2 was cotransfected. The histogram shows the mean + SD Firefly/*Renilla* ratio relative to Soak. dsCtl from four independent experiments (*n* = 12). ANOVA followed by Tukey’s post hoc test was used to detect significant differences between treatments. (**B**) Blocking of dsRNA-Cy3 internalization evaluated by fluorescence confocal imaging of single Z-sections. S2Xpress cells were incubated with anti-Hsc70-4 (80 ng/μl) or control IgG (80 ng/μl) for 1 hour. After washing with PBS, dsRNA-Cy3 was added to the growth medium for 40 min. dsRNA is shown in magenta, actin is shown in green, and nuclei are shown in blue. (**C**) Proposed model for dsRNA internalization. Our data suggest that Hsc70-4 acts as a co-receptor with a cell surface partner to bind and internalize extracellular dsRNA. The model was created using BioRender.com. Scale bars represent 5 μm. *P < 0.05; ****P < 0.0001.

Our results confirmed that Hsc70-4 is present at the cell surface of S2 cells and can bind dsRNA in vitro. We also showed that RNAi silencing of Hsc70-4 impairs dsRNA internalization and that this previously unknown role for Hsc70-4 is independent of its chaperone activity. We then hypothesized that Hsc70-4 could be acting as a cell surface receptor or co-receptor for exogenous dsRNA. To test this, we examined the effect of treating cells with an anti-Hsc70-4 antibody on dsRNA internalization using a luciferase/*Renilla* assay. S2Xpress cells were incubated with increasing concentrations of anti-Hsc70-4. The antibody was then removed, and the cells were washed with phosphate-buffered saline (PBS) and soaked with dsRNA (dsFluc or dsCtl). An unrelated immunoglobulin G (IgG) antibody was used as a control for nonspecific blocking. We observed that pretreatment of cells with anti-Hsc70-4 impaired luciferase activity compared to the untreated or IgG controls, indicating that dsRNA was unable to enter the cells (Fig. 5A). To visualize this effect, we used fluorescence confocal imaging in S2Xpress cells treated with anti-Hsc70-4 and then soaked with Cy3-labeled dsRNA. We observed that some anti-Hsc70-4–treated cells exhibited a punctate Cy3 pattern on the cell surface, indicating a complete blockage of dsRNA internalization (Fig. 5B). The observation that the pretreatment with anti-Hsc70-4 blocks dsRNA uptake but does not prevent dsRNA binding to the cell surface suggests that a second unknown partner is involved in the process. Together, our results support the role of Hsc70-4 as a co-receptor for the binding and internalization of environmental dsRNA (Fig. 5C).

## DISCUSSION

Insects use dsRNA internalization as a critical step in the systemic defense mechanism against viral infections and other pathogens (*4*, *9*, *10*). Upon detection of extracellular dsRNA, insect cells initiate receptor-mediated endocytosis, enabling interaction with intracellular RNAi machinery (*11*, *12*). This mechanism not only strengthens their antiviral response but also contributes to regulating endogenous gene expression and adapting to environmental challenges (*42*). Here, we sought to use *D. melanogaster* S2 cells as an in vitro model system to identify key cell membrane components involved in the uptake of extracellular dsRNA.

Our proteomics assays suggest that FBS deprivation drives an adaptation in S2 cells, evidenced by increased mitochondrial proteins at the plasma membrane. Previous studies show that mitochondria can migrate to the membrane, sometimes engaging with the extracellular environment, particularly under stress (*43*–*45*). Under nutrient-deficient conditions, such as FBS-free culturing, S2Xpress cells may undergo similar membrane adaptations without affecting proliferation or apoptosis. Therefore, with our model, we achieved the necessary resolution to examine a critical step in systemic immunity and identified potential 15 dsRNA binding proteins on the surface of S2 cells that were permissive to dsRNA internalization.

Among these, Hsc70-4 emerged as a key player in dsRNA internalization. Hsc70-4, a member of the highly conserved heat shock protein family, is known for diverse roles, including clathrin-mediated endocytosis, RNAi, and antiviral defense (*34*, *46*–*49*). Notably, Hsc70-4 was necessary for dsRNA internalization, whereas its homolog Hsc70-5 (52% amino acid identity) was not, underscoring a specific function for Hsc70-4. The predicted glycosylation site at the N terminus of Hsc70-4, which is not found in Hsc70-5, may confer this unique functionality (*50*).

Heat shock proteins that belong to the Hsp70 family are chaperones that have ATPase activity, and most of their cellular functions are mediated via ATP hydrolysis, which also allows for substrate binding. While Hsc70-4’s role in clathrin-mediated endocytosis is established (*31*), here, we provide evidence of a noncanonical role of Hsc70-4 at the cell surface. This finding is consistent with other studies in mammalian models showing the presence of heat shock proteins in the plasma membrane (*51*, *52*). In particular, it has been reported that Hsp70 translocates into the plasma membrane of human cell lines after stress and can integrate into artificial lipid bilayers (*51*). In addition, Hsp70 was found to bind to extracellular phosphatidylserine in tumor cells (*52*). Our data show that Hsc70-4 interacts with dsRNA and facilitates its internalization independently of ATP or chaperone activity. While intriguing, this observation aligns with previous findings suggesting that Hsc70-4 has membrane-deforming capabilities independent of its ATPase activity (*53*). Thus, membrane binding and functions in dsRNA internalization may offer unexpected insights into yet uncharacterized functions of Hsp70 family of proteins that do not rely on their ATPase activity.

Here, our findings point to Hsc70-4 as a potential dsRNA receptor or co-receptor. However, Hsc70-4 alone is likely insufficient for dsRNA uptake, as overexpression in S2naive cells did not induce internalization, hinting at roles that additional co-receptors or regulatory factors play. In addition, dsRNA continues to bind to the cell surface of S2Xpress cells even when Hsc70-4 is silenced, supporting our proposed model and indicating the involvement of a co-receptor. Given that Hsp70 family members are subject to a wide range of posttranslational modifications that regulate their function (*54*), it is also highly likely that specific posttranslational modifications on Hsc70-4 allow for this additional function at the cell surface. Comparative insights from mammalian systems further support this hypothesis. For instance, the mammalian ortholog HSPA8 serves as a surface co-receptor for viral entry in conjunction with other factors, such as CD163 (*55*). This dual role—binding extracellular ligands and facilitating endocytosis—parallels the proposed mechanism for Hsc70-4 in dsRNA internalization. In addition, the specificity for long dsRNA over siRNA (21 bp) (*12*) may reflect a requirement for cooperative binding by Hsc70-4 and an unidentified co-receptor.

As a pioneering study identifying Hsc70-4 as an extracellular dsRNA binding protein, this work lays the groundwork for further research into its structural and functional properties, potential co-receptors, and in vivo roles. We aim to address several key questions, including how broadly this mechanism applies to other systems, which specific adaptation endows S2Xpress cells with the ability to internalize dsRNA, and—because Hsc70-4 alone is insufficient—what additional component(s) complete this process. Possibilities include competing proteins, posttranslational modifications, or co-receptors. Despite these uncertainties, our findings reveal that in *Drosophila* cells, Hsc70-4 functions as a membrane protein having previously unrecognized biochemical traits that enable dsRNA binding and direct its uptake. This finding opens unexplored avenues for advancing RNAi technologies, with promising agricultural and economic benefits.

## MATERIALS AND METHODS

### Experimental design

Our goal was to find a dsRNA cell surface mediator(s) of dsRNA internalization in *Drosophila*. To this aim, we developed a cell model using S2 cells. Ultimately, this model allowed us to compare by proteomics the cell surface composition and presence of dsRNA binding proteins between cells that can internalize dsRNA versus cells that cannot. A selected list of candidates was tested by a high-content screen, and the results were validated by fluorescence, luciferase assays, and EMSA.

### Cells, plasmids, and antibodies

*D. melanogaster* Schneider 2 (S2naive) cells (Invitrogen) were cultured at 25°C in Schneider’s Insect Medium (Gibco), supplemented with 10% heat-inactivated FBS (Gibco), 2 mM l-glutamine (Gibco), penicillin (100 U/ml; Gibco), and streptomycin (100 mg/ml; Gibco). S2Xpress cells were cultured in Insect-XPRESS protein-free medium (Lonza, Belgium) supplemented with penicillin (100 U/ml) and streptomycin (100 mg/ml; Gibco). S2-H19 cells were cultured in Insect-XPRESS protein-free medium (Lonza, Belgium) supplemented with penicillin (100 U/ml), streptomycin (100 mg/ml; Gibco), and puromycin (8 μg/ml; QLL-44-08, InvivoGen). Expression of H19 was induced by supplementation with 4 μM cadmium chloride for 24 hours before staining. S2naive and S2Xpress cells were checked by next-generation sequencing of small RNAs for infection with CrPV, DAV, DCV, DXV, FHV, Nora virus, Sigma virus, American nodavirus, and *Drosophila* birnavirus. pMT/V5-HisB (Invitrogen) expressing either firefly or *Renilla* luciferase under the control of a copper-inducible promoter was previously generated. The following antibodies were used: anti-Cy3/Cy5 (ab52060, Abcam), anti-dsRNA J2 (10010500, SCICONS), anti-α-tubulin (T5168, Sigma-Aldrich), anti-Mouse IgG [horseradish peroxidase (HRP)] (ab6728, Abcam), anti-Mouse IgG (Alexa Fluor 488) (A11029, Invitrogen), anti-Mouse IgG (Alexa Fluor 555) (A21422, Invitrogen), anti-Rabbit IgG (HRP) (ab97051, Abcam) (A16096, Thermo Fisher Scientific), anti-Rabbit IgG (Alexa Fluor 488) (A11034, Invitrogen), and anti-Human-Hsc70 IgG (10654-1-AP, Proteintech). Polyclonal anti-Hsc70-4 was produced by GenScript by rabbit immunization with full-length protein. Polyclonal rabbit IgG control was produced by GenScript from nonimmunized rabbits. Anti-H19 was produced by immunization of mice with purified recombinant protein H19.

### Adaptation of S2 cells to Insect-Xpress medium

To generate the S2Xpress cells, S2naive cells were gradually adapted to the Insect-XPRESS protein-free medium. Briefly, S2naive cells were grown until confluency in a T25 flask in normal growth medium (Schneider’s Insect Medium, supplemented with 10% FBS) and then transferred into a T75 flask, adding 5 ml of Insect-Xpress medium. Four days after, 5 ml of Insect-XPRESS medium was added. At day 8, cells with medium were transferred into a T150 flask, and 15 ml of Insect-XPRESS medium was added. At day 11, 15 ml of cell suspension was transferred into a new T150 flask, and 15 ml of Insect-XPRESS medium was added. From this point forward, cells were adapted to the Insect-XPRESS medium and were passaged only using this medium.

### Cell growth and viability assay

To assess cell growth and viability in S2naive and S2Xpress cell lines, 2 × 10^6^ cells per well were seeded in triplicate in six-well plates. Cells were harvested and counted at 0, 24, 48, and 72 hours using 0.4% trypan blue dye (T10282, Invitrogen) with an automated cell counter (Countess II, Invitrogen). The absolute and relative numbers of live cells were recorded and plotted as log_2_ fold changes relative to the cell count at time 0 and as percentages of live cells, respectively.

### TUNEL assay

To assess the number of apoptotic cells, the DeadEnd Fluorometric TUNEL System kit (G3250, Promega) was used. S2naive and S2Xpress cells were seeded into an eight-chamber Nunc LabTek II plate previously coated with poly-l-lysine (P4832, Sigma-Aldrich). After 24 hours, the cells were washed with PBS, fixed in 4% paraformaldehyde (PFA) for 20 min, and permeabilized with 0.2% Triton X-100 in PBS for 5 min. TUNEL (terminal deoxynucleotidyl transferase–mediated deoxyuridine triphosphate nick end labeling) staining was carried out following the manufacturer’s instructions, with apoptotic nuclei appearing green and all cell nuclei counterstained with 4′,6-diamidino-2-phenylindole (DAPI). Vectashield H-1000 (Vector Laboratories, Burlingame, CA) was used as the mounting medium. Imaging was conducted using a Leica TCS SP5 confocal microscope at a 630× magnification. Five random images of single Z-sections per cell line were captured in each experiment. Image processing was performed in Fiji (*56*).

### dsRNA production

dsRNA was produced by in vitro transcription using the MEGAscript T7 Transcription kit (AM1334, Invitrogen) following the manufacturer’s guidelines. For dsRNAs targeting endogenous genes, cDNA produced with SuperScript II Reverse Transcriptase (100004925, Invitrogen) from S2Xpress RNA was used as a PCR template to amplify the target regions using primers flanked by the T7 promoter. For dsFLuc (firefly luciferase, GL3) and dsGFP, plasmids were used as templates (pMT-GL3 and pAc5.1-GFP). For dsCat (cathepsin-I) used in EMSAs, cDNA from aphids was used as a template. dsRNA concentration was quantified on a Qubit 3 fluorometer. All dsRNAs produced against candidates were 450 to 600 bp in length (dsGL3, 557 bp; dsGFP, 714 bp; dsCG6647, 508 bp; dsCat, 398 bp). The full list of primers can be found in table S5.

### Nucleic acid labeling with Cy3

The Silencer siRNA Labeling kit with Cy3 (AM1632, Invitrogen) was used to label dsRNA (dsFLuc, dsGFP, dsCat, and dsCG6647), DNA (FLuc and Cat), or siRNA [glyceraldehyde-3-phosphate dehydrogenase (GAPDH), from the kit] with Cy3 following the kit’s protocol. Labeling was confirmed by an electrophoretic shift of the purified product compared to unlabeled probe on a 1.5% agarose gel and by immunofluorescence with the anti-dsRNA antibody, J2 (fig. S1, C and D).

### Fluorescence microscopy

S2naive and S2Xpress cells were seeded in an eight-chamber Nunc LabTek II that had previously been coated with poly-l-lysine (P4832, Sigma-Aldrich). Cells were then incubated for 24 hours. For silencing experiments, cells were transfected with dsRNA using the Effectene Transfection reagent (301427, Qiagen) and incubated for 72 hours. For endocytosis inhibition experiments, Dynasore (D7693, Sigma-Aldrich) was added to a final concentration of 100 μM for 10 to 60 min at 25°C. For the antibody-blocking assay, anti-Hsc70-4 (GenScript) or IgG control (GenScript) was added to a final concentration of 80 ng/μl for 1 hour at 25°C; cells were washed with PBS and growth medium was added. dsRNA, DNA, or siRNA labeled with Cy3 (to a final concentration of 0.76 nM) was added, and cells were incubated at 25°C for 30 to 40 min. Cells were washed with PBS, fixed in 4% PFA for 20 min, and blocked/permeabilized in 2% bovine serum albumin (BSA)-0.2% Triton X-100. Actin was visualized with Oregon Green 488 Phalloidin (O7466, Invitrogen). For immunofluorescence with J2 and anti-Cy3 antibodies, blocking/permeabilization was done with 10% normal goat serum-0.2% Triton X-100 followed by an overnight incubation at 4°C with a primary antibody (J2, 1:500; anti-Cy3, 1:500). Slides were incubated with a secondary antibody (anti-Mouse IgG-Alexa Fluor 488, 1:1000) for 1 hour, and no actin staining was performed. For immunofluorescence to detect Hsc70-4 under permeabilized conditions, staining was done as previously described using anti-Hsc70-4 (1:100) as a primary antibody and anti-Rabbit IgG-Alexa Fluor 488 (1:1000) as a secondary antibody. For nonpermeabilized conditions, blocking and antibody incubation steps were done before fixation at 4°C. Cells were blocked in 10% normal goat serum and incubated with a primary antibody (30 min) and secondary antibody (30 min) at 4°C. Cells were then fixed with 4% PFA for 20 min. For immunofluorescence staining to detect H19 under permeabilized and nonpermeabilized conditions, induced S2-H19 cells were stained as previously described using anti-H19 (1:500) and anti-Mouse IgG-Alexa Fluor 488 (1:1000) as primary and secondary antibodies, respectively. Actin was visualized with Alexa Fluor 555 Phalloidin (A34055, Invitrogen) (except when dsRNA-Cy3 was used). Nuclei were counterstained with DAPI. Vectashield H-1000 (Vector Laboratories, Burlingame, CA) was used as mounting medium. For rHsc70-4 experiments, the coding region for Hsc70-4 (CG4264) was cloned into pAc5.1/V5-His A (Invitrogen). Assembly was done with NEBuilder HiFi DNA Assembly Master Mix (E2621L, New England Biolabs). Correct insertion of the amplicon was confirmed by sequencing. Cells were transfected with pAc5.1-rHsc70-4-V5/His and incubated for 48 hours before soaking with dsRNA-Cy3 and posterior fixing. Staining was done as previously described. An anti-V5 Tag primary antibody was used to detect rHsc70-4-V5/His at 1:1000 dilution. Nuclei were counterstained with DAPI. Imaging was done on a Leica TCS SP5 confocal microscope at a 630× magnification as single Z-sections, and images were processed in Fiji (*56*).

### Luciferase assays

Cells were seeded on 96-well culture plates and transfected with plasmids pMT-GL3 (firefly luciferase, 12 ng per well) and pMT-*Renilla* (*Renilla* luciferase, 3 ng per well), and with dsRNA (10 ng per well) when indicated, using the Effectene Transfection reagent (301427, Qiagen). After 24 hours, the medium was changed, and dsFLuc or dsCtl (dsGFP) (50 ng per well, or the indicated amounts) was added (soaking) to allow internalization and posterior silencing of FLuc. After 24 hours, plasmid expression was induced by adding 10 mM CuSO_4_. The next day, cells were lysed, and measurement of firefly and *Renilla* luciferase activity was performed using the Dual-Luciferase Reporter Assay System (E1960, Promega) in a GLOMAX microplate luminometer. Firefly luciferase values were normalized to *Renilla* luciferase values. For analysis of the effect of the medium on internalization, the medium was changed to the indicated medium before soaking and then soaking was performed for 4 hours. Following soaking, the medium was changed back to the appropriate growth medium for each cell type. To test the silencing capacity of Cy3-labeled dsRNA, Cy3-labeled dsFLuc was used. For silencing experiments, plasmids were cotransfected with target dsRNA (dsHsc70-4 and dsAgo2) for 3 days before soaking. For antibody-blocking experiments, cells were incubated with anti-Hsc70-4 (40, 60, and 80 ng/μl) (GenScript) or IgG control (80 ng/μl) (GenScript) diluted in Insect-XPRESS medium. After 1 hour, cells were washed with PBS, the medium was replaced, and dsRNAs were added. For JG-98 experiments, cells were incubated with JG-98 (0.125, 0.25, 0.5, and 1 μM) (TA9H97BAEC49, Merck) or DMSO (1 μM) (D2650, Sigma-Aldrich) for 20 min before soaking.

### Purification and identification of cell surface proteins

S2naive and S2Xpress cells were seeded in T75 flasks and incubated until the next day when they were collected and counted. A total of 2.4 ×10^7^ cells was diluted in 15 ml of growth medium. Biotinylation and purification of biotinylated proteins were done following the Pierce Cell Surface Biotinylation and Isolation Kit’s (A44390, Thermo Fisher Scientific) protocol. Samples without biotin were used as negative controls to identify nonspecific protein precipitation. Samples were eluted in 200 μl of elution buffer. Fifty microliters of purified proteins was used for proteomic analysis, and a separate 50-μl aliquot was used for gel analysis. Laemmli SDS sample buffer (6×; J60660, Alfa Aesar) (with β-mercaptoethanol) was added to the samples, and the samples with Laemmli buffer were boiled for 5 min at 100°C. For proteomic analysis, samples were loaded on a 7.5% SDS–polyacrylamide gel electrophoresis (SDS-PAGE) gel (Bio-Rad) before in-gel proteolysis. Briefly, gel bands corresponding to proteins were excised and washed and then the proteins were reduced and alkylated [dithiothreitol (DTT; final concentration, 10 mM), 2 hours, 37°C, iodoacetamide (final concentration, 50 mM), 30 min in the dark at room temperature]. After dehydration, protein proteolysis was done with 50 ng of LysC-trypsin (Promega) at 37°C overnight. The resulting proteolytic peptides were extracted from the gel by incubating twice for 15 min in 100 μl of 1% aq. trifluoroacetic acid and sonication, followed by one incubation of 15 min at 37°C in 50 μl of acetonitrile (ACN). Peptides were desalted using C18-filled tips (Ziptip C18, Millipore), eluted in 10 μl of H_2_O/CAN 1:1 (v/v) 0.1% trifluoroacetic acid, dried, and dissolved in 8 μl of solvent A. The digest (6 μl of peptides) was injected on a capillary reversed-phase column (C18 Acclaim PepMap100Å, 75-μm inner diameter, 50-cm length; Thermo Fisher Scientific) at a flow rate of 220 nl/min, with a gradient of 2 to 40% solvent B in solvent A in 60 min [solvent A, H_2_O/ACN/FA 98:2:0.1 (v/v/v); solvent B, H_2_O/ACN/FA 10:90:0.1 (v/v/v)]. MS analysis was performed on a Q Exactive HF mass spectrometer (Thermo Fisher Scientific) with a top 10 data-dependent acquisition method: MS resolution of 70,000 and mass range of 400 to 2000 Da, followed by MS/MS on the 10 most intense peaks at a resolution of 17,500, with a dynamic exclusion for 10 s. Raw data were processed using Proteome Discoverer 2.4 (Thermo Fisher Scientific). The database search was done with the Mascot search engine (Matrix Science Mascot 2.2.04) on a *D. melanogaster* protein databank (20,986 entries). The SwissProt databank 2020_05 (563,552 entries) was used to assess contamination with human proteins. The following parameters were used: MS tolerance, 10 ppm; MS/MS tolerance, 0.02 Da; tryptic peptides; up to two miscleavages; partial modifications, carbamidomethylation C, oxidation (M), and deamidation (NQ). Proteins identified by at least two high-confidence peptides (false discovery rate <0.1%) were validated. Further analysis of the identified proteins was done on FunRich software (*57*). For SDS-PAGE, samples were loaded on a 4 to 15% polyacrylamide gradient gel. After electrophoresis, the gel was fixed with 40% ethanol-10% acetic acid-50% H_2_O and silver staining was performed as previously described (*58*).

### IP of dsRNA binding proteins

S2Xpress cells were seeded on 100-mm culture plates (1 × 10^7^ cells/10 ml per plate) and incubated for 24 hours at 25°C. Then, the medium was changed, the endocytosis inhibitor Dynasore (D7693, Sigma-Aldrich) was added (final concentration, 100 μM), and cells were incubated for 1 hour at 25°C. Next, dsRNA (dsFLuc) labeled with Cy3 was added (10 μg per plate) and soaking was performed for 45 min at 25°C. For negative control plates, unlabeled dsRNA was used. Following soaking, plates were washed in cold PBS and UV cross-linked (254 nm) in 3 ml of PBS (without Mg/Ca) at 300 mJ/cm^2^ on ice in a Stratalinker UV 1800 cross-linker. Cells were scraped from the plates, pelleted by centrifugation, and lysed in SDS lysis buffer [0.5% SDS, 50 mM tris, 1 mM EDTA, 1 mM DTT, pH 8, and 1× protease inhibitor (11 873 580 001, Roche)] at 65°C for 5 min. Next, radioimmunoprecipitation assay (RIPA) correction buffer was added (62.5 mM tris, 1.25% NP-40 substitute, 0.625% sodium deoxycholate, 2.25 mM EDTA, 187.5 mM NaCl, pH 8, and 1× protease inhibitor), and samples were passed through QIAshredder columns (79654, Qiagen) twice, following the manufacturer’s recommendations. Following this, the samples were incubated with 4 μg of antibody (anti-Cy3) overnight at 4°C on a rotator. The next day, the Dynabeads Protein A Immunoprecipitation Kit (10006D, Invitrogen) was used to pull down dsRNA-protein complexes binding to the antibody, following the kit’s protocol. Briefly, samples were incubated with the magnetic beads for 1 hour at room temperature. Next, samples were diluted and washed five times with RIPA buffer [50 mM tris, 1% NP-40 substitute, 0.5% sodium deoxycholate, 0.1% SDS, 2 mM EDTA, 150 mM NaCl, pH 8, and 1× protease inhibitor (911 873 580 001, Roche)] before elution. To elute proteins, the samples were first treated with ribonuclease III for 1 hour at 37°C, and supernatants were collected. Next, the beads were treated with 6× Laemmli SDS sample buffer (J60660, Alfa Aesar) (with 10% β-mercaptoethanol) and boiled for 5 min at 100°C and the supernatants were collected. Proteomics analysis was done as detailed in the previous section. Samples collected after ribonuclease III treatment had only a few proteins, indicating that the elution was not effective. Thus, only samples collected after boiling in sample buffer were considered. Duplicates were performed for each treatment. Negative control samples were used to discard proteins that were nonspecifically precipitated. Analysis of the identified proteins was done using FunRich software (*57*).

### High-content imaging screen

Cells were seeded on black CellCarrier Ultra microplates (6055302, PerkinElmer) and transfected with dsRNA (10 ng per well) targeting the candidates using the Effectene Transfection reagent (301427, Qiagen). dsCtl and Dynasore (D7693, Sigma-Aldrich) wells were transfected with dsCtl (dsFLuc). After 72 hours of incubation, the medium was changed and Dynasore was added to corresponding wells (final concentration, 100 μM) for 10 min at 25°C. Next, dsRNA (30 ng per well; dsFLuc) labeled with Cy3 was added. Cells were then incubated for 30 min at 25°C, fixed in 4% PFA for 20 min, and blocked/permeabilized in 2% BSA-0.2% Triton X-100 for 30 min. The cytoplasm was visualized with DiO dye (from Vybrant Multicolor Cell-Labeling Kit; V22889, Molecular Probes), and nuclei were counterstained with DAPI. Imaging was performed on single Z-sections on an Opera Phenix microscope (PerkinElmer) at a 630× magnification. Columbus software was used to design a script and analyze the images. The script was designed to quantify the median intensity of Cy3 and the number of spots of Cy3 in the cytoplasm per well. Further analysis was done using GraphPad Prism 9. For the median fluorescence intensity, negative control wells were used to subtract baseline intensity values.

### RT-qPCR

Cells were seeded in 24-well plates and incubated for 24 hours. RNA extraction was performed with TRIzol reagent (15596026, Ambion), and RNA concentration was quantified using a NanoDrop One (Thermo Fisher Scientific). Equal amounts of RNA were treated with RQ1 deoxyribonuclease (M610A, Promega) and were used to produce cDNA with the Maxima H Minus First Strand cDNA Synthesis Kit (K1682, Thermo Fisher Scientific) using random primers. qPCR was done with Luminaris Color HiGreen qPCR Master Mix, low ROX (K0374, Thermo Fisher Scientific) on a QuantStudio 7 Flex Real-Time PCR System (Applied Biosystems). Rp49 was used as a housekeeping gene. Δ*C*_t_ values were obtained by subtracting the *C*_t_ value for Rp49 from the *C*_t_ value of the corresponding gene, and ΔΔ*C*_t_ values were obtained by further subtracting the geometric mean Δ*C*_t_ value for the control condition (S2naive). Results are shown as a fold change relative to S2naive. Primer sequences can be found in table S5.

### RT-PCR to confirm silencing

To confirm silencing of Hsc70-4 (CG4264) by dsHsc70-4, cells were transfected with dsHsc70-4 with the Effectene Transfection reagent (301427, Qiagen) and incubated for 72 hours. For the control condition, cells were transfected with dsCtl (dsFLuc). RNA was extracted, and cDNA was produced as previously described, with the modification that oligo(dT)_18_ primers were used. PCR was performed with DreamTaq DNA Polymerase (EP0702, Thermo Fisher Scientific) using primers flanking the dsRNA targeting region. PCR products were loaded on a 1% agarose gel with ethidium bromide. After electrophoresis, the gel was developed on a Gel Doc XR+ Imaging System (Bio-Rad). Rp49 was used as a loading control. Primers sequences can be found in table S5.

### Western blot

To confirm the silencing of Hsc70-4 by dsHsc70-4 by Western blot, S2Xpress cells were seeded in six-well plates and transfected with dsHsc70-4 or dsCtl, as described above. For quantification of Hsc70-4 in S2naive and S2Xpress cells, no transfection was done. In both cases, after 24 hours, cells were washed with cold PBS and proteins were extracted with RIPA buffer [50 mM tris, 1% NP-40 substitute, 0.5% sodium deoxycholate, 0.1% SDS, 2 mM EDTA, 150 mM NaCl, pH 8, and 1× protease inhibitor (11 873 580 001, Roche)]. Samples were incubated on ice for 20 min and centrifuged at 13,000*g* for 20 min at 4°C. Supernatants were collected. To confirm expression of Hsc70-4, equal volumes of samples were boiled in XT Sample Buffer (161-0791, Bio-Rad) with β-mercaptoethanol for 5 min and loaded on a 4 to 20% Mini-PROTEAN TGX Stain-Free Protein Gel (4568094, Bio-Rad). PageRuler Prestained Protein Ladder (26616, Thermo Fisher Scientific) was used as a molecular weight ladder. After electrophoresis, the gel was activated and then transferred for 30 min to a Trans-Blot Turbo Mini 0.2-μm nitrocellulose membrane (1704158, Bio-Rad) on a Trans-Blot Turbo transfer system (Bio-Rad). Total proteins were visualized by UV on a Gel Doc XR+ Imaging System (Bio-Rad). Membranes were blocked with 5% nonfat dry milk in PBS-0.05% Tween 20 and incubated overnight with a primary antibody at 4°C (anti-Hsc70-4, 1:1000; anti-α-tubulin, 1:5000). Washes were done with PBS-0.05% Tween 20. Membranes were incubated for 1.5 hours with a secondary antibody [anti-Rabbit IgG (HRP), 1:5000; anti-Mouse IgG (HRP), 1:5000], developed with SuperSignal West Pico PLUS Chemiluminescent Substrate (34580, Thermo Fisher Scientific), and imaged on a ChemiDoc MP Imaging System (Bio-Rad). The protein concentration in the samples was quantified with the Pierce BCA Protein Assay Kit (23227, Thermo Fisher Scientific), and an equal amount of proteins was loaded on the gel. α-Tubulin was used as a loading control.

### IP with the anti-Hsc70-4 antibody

To confirm the specificity of the anti-Hsc70-4 antibody, an IP assay was conducted using the Dynabeads Protein A Immunoprecipitation Kit (10006D, Invitrogen). S2naive and S2Xpress cells were seeded onto 100-mm culture plates at a density of 1 × 10^7^ cells/10 ml per plate and incubated at 25°C. After 24 hours, the cells were washed with cold PBS, and proteins were extracted using RIPA buffer. The supernatants were collected, and the total protein content was quantified using the Pierce BCA Protein Assay Kit (23227, Thermo Fisher Scientific). The protein samples (input) were precleared by incubation with magnetic beads for 1 hour at 4°C. Subsequently, the precleared samples were incubated with either anti-Hsc70-4 or control antibody for 1 hour. The precleared + antibody samples were then incubated with fresh magnetic beads overnight at 4°C. The following day, the supernatant was discarded, and proteins bound to the beads were eluted with the kit buffer. The beads were subsequently resuspended in 2× Laemmli sample buffer (42526, Serva) and boiled. The boiled supernatant was recovered by centrifugation and analyzed by LC-MS/MS. Duplicates were run for each treatment, and negative control samples were included to eliminate proteins not specifically precipitated. A label-free quantification method was used (based on the area under the peaks of the peptides) to compare the relative abundancy of proteins identified in the anti-Hsc70 and control antibody samples. We considered a ratio of above 10 significant. If a protein was not identified in the control sample, this ratio will be 100. Because Western blot analysis showed that anti-Hsc70-4 only detects proteins of ~70 kDa, everything else was considered unspecific coelution or complex precipitation. Although there are a few proteins detected of about 70 kDa, Hsc70-4 was by far the one detected with more confidence for all values for both cells lines (table S3), confirming the specificity of an antibody.

### Proteomics of total membrane extracts

S2naive and S2Xpress cells were grown in 3-liter cultures. After 48 hours, cells were collected in 50 mM Hepes/tris base (pH 7.4), 50 mM NaCl buffer supplemented with 30% glycerol, 1 mM EDTA, 6 mM phenylmethylsulfonyl fluoride, 10 mM tris(2-carboxyethyl)phosphine, and 1:2000 (v/v) dilution of cOmplete Protease Inhibitor Cocktail (Sigma-Aldrich). Solubilization was performed for 20 min in a rotor at 4°C and disrupted in a cell homogenizer (EmulsiFlex-C5, Avestin) after three runs at 103.4 MPa. Disrupted cell homogenates were clarified by centrifugation at 4500*g* for 25 min, and membranes were recovered by ultracentrifugation at 40,000*g* for 90 min at 4°C. Membranes were further homogenized using a dounce and then washed with the abovementioned buffer before a second round of ultracentrifugation at 40,000*g* for 90 min at 4°C to recover membranes that were further snap frozen in liquid N_2_ and stored at −80°C until use. Membrane solubilization was done by thawing out first in 50 mM Hepes/tris base (pH 7.4), 50 mM NaCl, 20% glycerol, 10 mM tris(2-carboxyethyl)phosphine, 6 mM phenylmethylsulfonyl fluoride, and 30 mM cOmplete Protease Inhibitor Cocktail (Sigma-Aldrich) before supplementing the membrane homogenate with 2% dodecanoyl sucrose (drop by drop with agitation in ice). Solubilization was performed for 1 hour at 4°C with rotation. Insoluble material was first removed by ultracentrifugation (1 hour at 40,000 rpm). Last, solubilized membranes were ultracentrifuged at 70,000 rpm for 1 hour at 4°C and supernatants were snap frozen in liquid N_2_ and stored at −80°C at 0.3 g of membranes ml^−1^. Membranes were run in 10% SDS-PAGE gel before in-gel proteolysis. Proteomics analysis was done by LC-MS/MS in duplicate for each cell line. For the analysis, proteins found in both replicates for each cell line were considered. Duplicated or nonexistent accession numbers (records removed from UniProt) were removed. Comparisons were done with FunRich software (*57*).

### Membrane lipid strip assay

rHsc70-4 protein was produced by GenScript by expression of the recombinant protein with a 6× His tag in *Escherichia coli*. The protein was purified with Ni resin. Recombinant human Hsc70 expressed in *E. coli* was used as a control. Membrane strips (Echelon Bio-Sciences, P-6002) were blocked with tris-buffered saline with Tween 20 (TBS-T) containing 3% BSA for 1 hour. Strips were incubated with protein (1 μg/ml) for 1 hour and washed with TBS-T. Strips were incubated with a primary antibody (anti-Hsc70-4, 1:8000; anti-Human-Hspa8, 1:8000 in blocking buffer) for 1 hour, washed with TBS-T, and incubated with a secondary antibody [anti-Rabbit IgG (HRP), 1:5000] for 1 hour. Proteins and antibody dilutions were prepared in blocking buffer. Incubations were done at room temperature. Strips were revealed with Amersham ECL (RPN2109, Amersham) and imaged on a ChemiDoc Imaging System (Bio-Rad).

### Electrophoretic mobility shift assay (EMSA)

For EMSA experiments, dsRNA (dsCat, dsCG6647, and dsFLuc of 50, 100, 250, or 500 bp), dsDNA (Cat), or siRNA (GAPDH) labeled with Cy3 were used as probes. The labeled probe (0.76 nM) was incubated with different concentrations of the rHsc70-4 protein (GenScript) in binding buffer [25 mM tris, 50 mM NaCl, BSA (0.1 mg/ml), 31.25 mM DTT, and tRNA *E. coli* (0.625 mg/ml); 20-μl reaction] for 30 min at 25°C. For nucleotide substrate assays, the labeled probe (0.76 nM) was incubated with rHsc70-4 protein (2 μM) and increasing concentrations of ATP-γ-S (11162306001, Sigma-Aldrich), ADP (A2754, Merck), or AMP-PNP (10102547001, Merck) in PBS. Native loading buffer was added after incubation (100 mM tris, 10% glycerol, and 0.0025% bromophenol blue, pH 8). For competition assays, 10 times more of the same unlabeled dsRNA was added to the reaction mix. EMSA gels (native 4% polyacrylamide and 0.5× tris-borate EDTA) were prerun at 200 V on ice before loading samples in 0.5× tris-borate EDTA buffer. Samples were loaded and ran at 200 V on ice. Gels were imaged by fluorescence detection on a Typhoon FLA 9000 (GE Healthcare).

### Statistical analysis

Data are represented as the means ± SD. Statistical analyses were done in GraphPad Prism version 9 (GraphPad Software, CA). Two-tailed unpaired *t* tests (two groups) or one-way analyses of variance (ANOVAs; three or more groups) followed by Dunnett or Tukey’s post hoc tests were used to detect significant differences between groups. *P* values <0.05 were considered significant. Normality and homoscedasticity were assumed for the data. Whenever these criteria were not met, nonparametric Welch’s ANOVA tests (three or more groups) followed by Dunnett’s T3 post hoc tests were used. For cellular component analysis of proteomic results, a hypergeometric test was done using FunRich software (*57*).
