# Supplementary material for: Hsc70-4: An unanticipated mediator of dsRNA internalization in Drosophila
Source: Sci Adv. 2025 May 16;11(20):eadv1286. doi: 10.1126/sciadv.adv1286 (PMC12083535; doi:10.1126/sciadv.adv1286)
Supplement: Supplementary file 1 — Figs. S1 to S5 Legends for tables S1 to S5 [file sciadv.adv1286_sm.pdf]

Science Advances
AAAS

Supplementary Materials for

Hsc70-4: An unanticipated mediator of dsRNA internalization in Drosophila

Sabrina J. Fletcher *et al.*

Corresponding author: Maria-Carla Saleh, carla.saleh@pasteur.fr; Shaeri Mukherjee, shaeri.mukherjee@ucsf.edu

Sci. Adv. **11**, eadv1286 (2025)
DOI: 10.1126/sciadv.adv1286

The PDF file includes:

Figs. S1 to S5
Legends for tables S1 to S5

Other Supplementary Material for this manuscript includes the following:

Tables S1 to S5

Fig. S1.

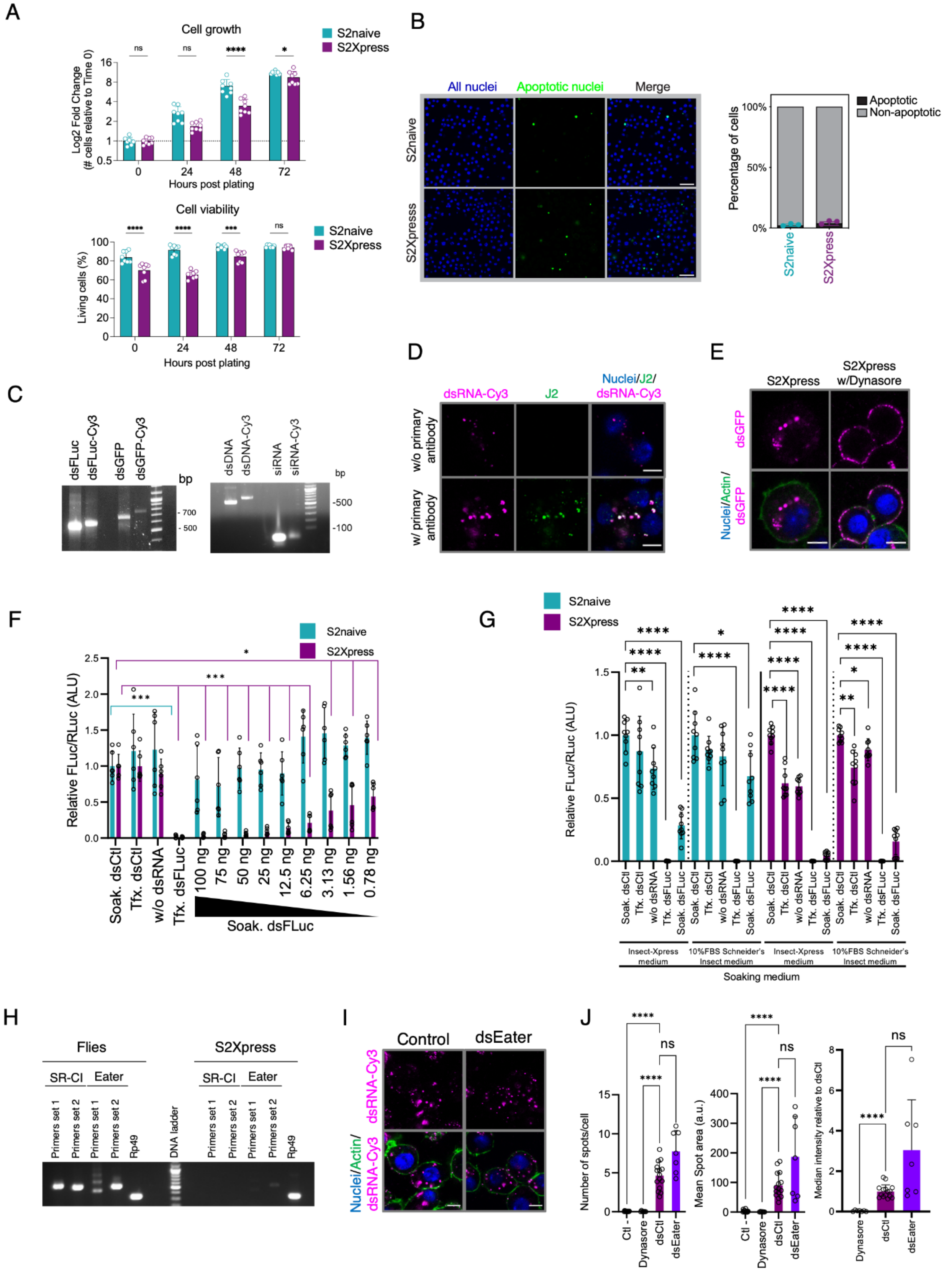
A
Cell growth
S2naive
S2Xpress
Log2 Fold Change (# cells relative to Time 0)
Hours post plating
Cell viability
Living cells (%)
B
All nuclei
Apoptotic nuclei
Merge
S2naive
S2Xpresss
Percentage of cells
Apoptotic
Non-apoptotic
C
dsFLuc
dsFLuc-Cy3
dsGFP
dsGFP-Cy3
bp
dsDNA
dsDNA-Cy3
siRNA
siRNA-Cy3
D
dsRNA-Cy3
J2
Nuclei/J2/dsRNA-Cy3
w/o primary antibody
w/ primary antibody
E
S2Xpress
S2Xpress w/Dynasore
dsGFP
Nuclei/Actin/dsGFP
F
Relative FLuc/RLuc (ALU)
Soak. dsCtl
Tfx. dsCtl
w/o dsRNA
Tfx. dsFLuc
Soak. dsFLuc
G
Insect-Xpress medium
10%FBS Schneider's Insect medium
Soaking medium
H
Flies
S2Xpress
SR-CI
Eater
Primers set 1
Primers set 2
Rp49
DNA ladder
I
Control
dsEater
dsRNA-Cy3
Nuclei/Actin/dsRNA-Cy3
J
Number of spots/cell
Mean Spot area (a.u.)
Median intensity relative to dsCtl
Ctl -
Dynasore
dsCtl
dsEater

Fig. S1. S2 model to study dsRNA internalization. (**A**) Cell growth rate (top) and viability (bottom) of S2naive and S2Xpress cells at 0, 24, 48 and 72 hours after plating. Cells were counted with trypan blue. Data are from 3 independent experiments (n=9) and compared by ANOVA Šidák. (**B**) Confocal imaging of single Z-sections of TUNEL assay in S2naive and S2Xpress cells to measure apoptosis (left). Apoptotic nuclei are in green and all nuclei are in blue. The scale bar represents 30 µM. Data from 3 independent experiments with 5 random images for each cell line were pooled and compared by Student's t-test (right). (**C**) Agarose gel electrophoresis of Cy3-labeled and unlabeled dsRNA (dsFLuc and dsGFP) (left), dsDNA and siRNA (right). Image contrast was adjusted for improved visualization. (**D**) Immunofluorescence of single Z-sections of S2Xpress cells with the anti-dsRNA antibody, J2 (green) to confirm that labeling of dsRNA (dsGL3) with Cy3 (magenta) was efficient and specific. Nuclei are in blue. Confocal images showed complete co-localization between Cy3 signal and J2 signal. (**E**) Cy3-labeled dsRNA corresponding to the sequence of GFP (dsGFP, magenta) was used to confirm that uptake of dsRNA by S2Xpress cells was not sequence-dependent. Soaking with dsRNA was done as in (D). Actin is in green and nuclei are in blue. For Dynasore experiments, Dynasore was added for 20 min before soaking with dsRNA. Images of single Z-sections were taken. (**F**) Dose-dependent silencing of Firefly luciferase. Cells were transfected as in (Fig. 1C) and the indicated amounts of dsRNA were added during soaking. Data are from 2 independent experiments (n=6) and indicate mean + SD of Firefly/Renilla ratio relative to Soak. dsCtl (Welch's ANOVA test). (**G**) Effects of soaking medium on dsRNA internalization. Experiments were done as in (Fig. 1C) using the indicated soaking medium for 4 h. Data are from 3 independent experiments (n=9) and indicate mean + SD of Firefly/Renilla ratio relative to Soak. dsCtl (Welch's ANOVA test). (**H**) Agarose gel electrophoresis of RT-PCRs product used as templates for in vitro transcription of dsRNA targeting SR-CI and Eater. Two sets of primers were used for each receptor. cDNA from w1118 flies was used a positive control and Rp49 as PCR control. We were not able to amplify SR-CI when cDNA from S2Xpress cells was used for PCR. (**I**) Confocal imaging of single Z-sections of S2xpress cells transfected with dsEater or not transfected (Control) for 24 h prior to soaking with 30 ng of Cy3-labeled dsRNA (dsFLuc, magenta) for 40 min. Actin is in green and nuclei are in blue. (**J**) High content imaging was used to test the effect of silencing Eater on the uptake of dsRNA. S2Xpress cells were transfected with 10 ng of dsEater to silence the receptor for 72 h. dsCtl and Dynasore wells were transfected with nonspecific dsRNA (dsFLuc). For Dynasore wells, Dynasore was added for 10 min prior to soaking with 30 ng of nonspecific Cy3-labeled dsRNA (dsFLuc) for 30 min. Plates were imaged on an Opera Phenix High content microscope. Histograms show mean + SD of the number of Cy3 spots/cell, mean spot area, and median Cy3 intensity compared to the control condition (dsCtl). Data are from 2 independent experiments pooled together (Ctl- n=15; Dynasore n=7; dsCtl n=15, dsEater n=7). For median intensity, Ctl- values were subtracted from the other conditions. Values are shown relative to dsCtl. Welch's ANOVA tests were used to detect significant differences compared to the control condition (dsCtl). Scale bars from (D), (E) and (I) represent 5 µM. *$p<0.05$; **$p<0.01$; ***$p<0.001$; ****$p<0.0001$.

Fig. S2.

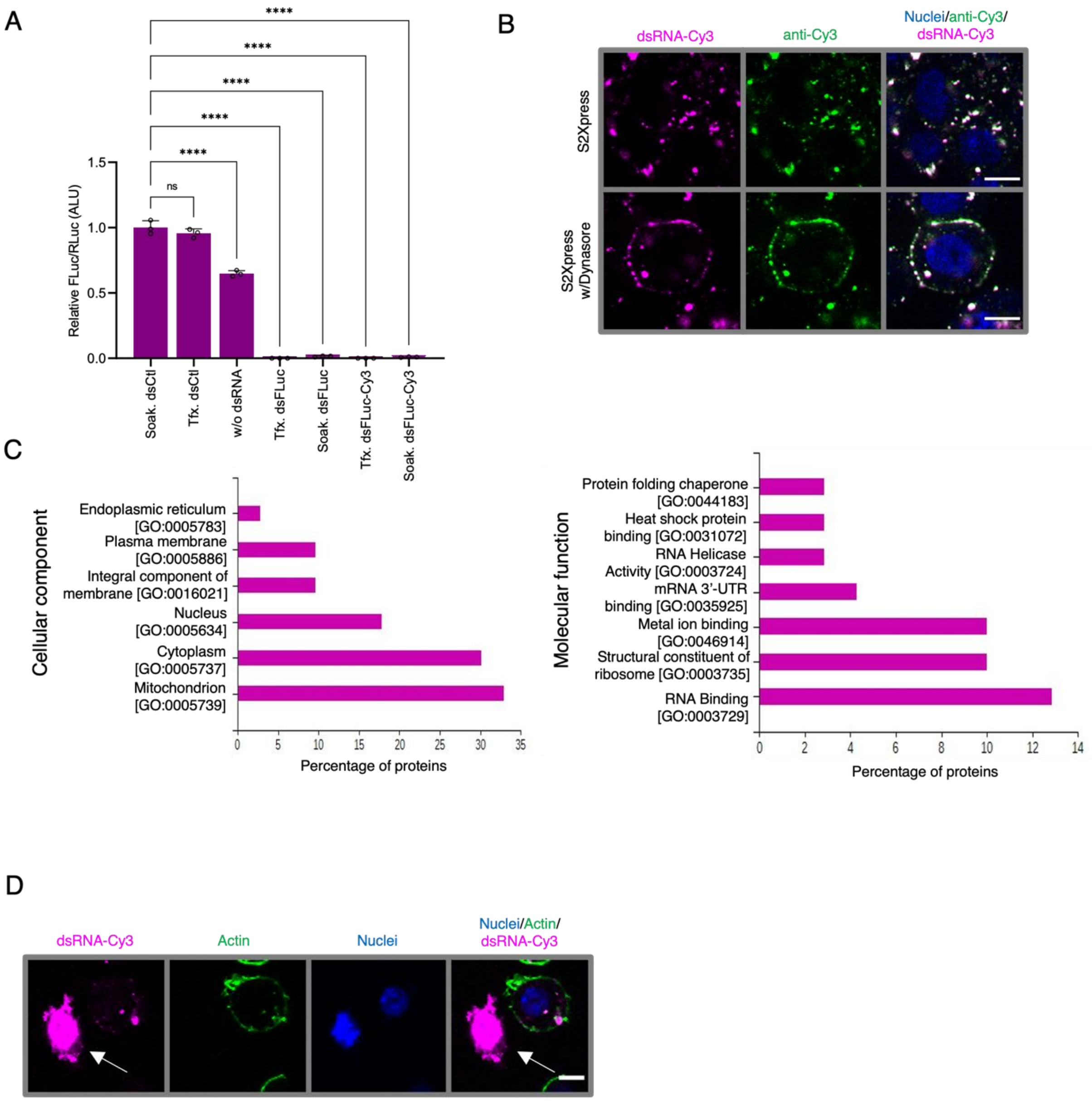

Fig. S2. Identification of cell surface dsRNA-binding proteins. (**A**) Silencing of Firefly luciferase by internalized Cy3-labeled dsRNA in S2Xpress cells. The experiment was done as in (Fig. 1C). The data show that Cy3-labeling does not affect the silencing capacity of dsRNA-FLuc. (**B**) Immunofluorescence with an anti-Cy3 antibody was performed to check specificity of antibody to be used for the IP of dsRNA binding proteins from S2Xpress cells. Immunofluorescence was performed with an anti-Cy3 antibody (green). Nuclei are in blue. Complete co-localization of dsRNA-Cy3 (magenta) and anti-Cy3, even when Dynasore was added, confirmed that this antibody is highly specific and thus a good option for IP. Images of single Z-sections were taken. (**C**) Cellular component and molecular function analysis of

proteins identified by IP of dsRNA-Cy3-protein complexes from S2Xpress cells (Fig. 1G). Cellular component and molecular function analyses were done with the FunRich software (*30*). (**D**) Visualization of the internalization of dsRNA-Cy3 (magenta) was performed as in (B) with minor modifications. Actin is in green and nuclei in blue. The arrow indicates a dying/dead cell with high Cy3 signal. dsRNA seems to bind to these death nuclei/cells, resulting in the immunoprecipitation of nuclear proteins. Confocal images were taken as single Z-sections at 630x magnification. Scale bars represent 5 μM. *p<0.05; **p<0.01; ***p<0.001; ****p<0.0001.

Fig. S3

A

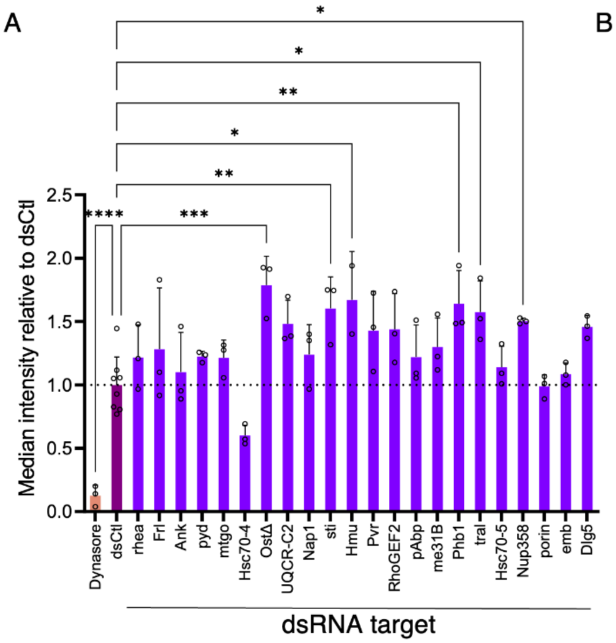

B

Treatment	Number of spots/cell p-value vs. dsCtl	Mean Spot area p-value vs. dsCtl
Ctl -	<0.0001	0.0009
Dynasore	<0.0001	0.0408
rhea	0.2393	0.9451
Frl	0.9891	0.9992
Ank	0.9997	>0.9999
pyd	0.8137	0.9147
mtgo	0.7128	0.6142
Hsc70-4	0.0004	0.1854
OstΔ	0.0003	<0.0001
UQCR-C2	0.0481	0.0632
Nap1	0.5229	0.7109
sti	0.0363	0.0009
Hmu	0.0228	0.0023
Pvr	0.1639	0.0288
RhoGEF2	0.3467	0.1077
pAbp	0.5278	0.5679
me31B	0.2166	0.5730
Phb1	0.0050	0.0003
tral	0.0134	0.0056
Hsc70-5	0.9992	0.9999
Nup358	0.0021	0.0003
porin	0.9949	0.8312
emb	0.1499	0.3955
Dlg5	0.0251	0.0206

C

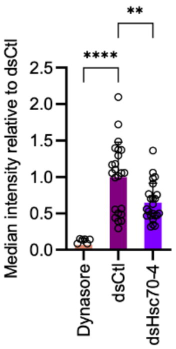

D

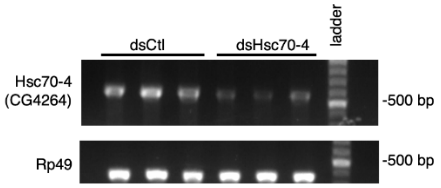

E

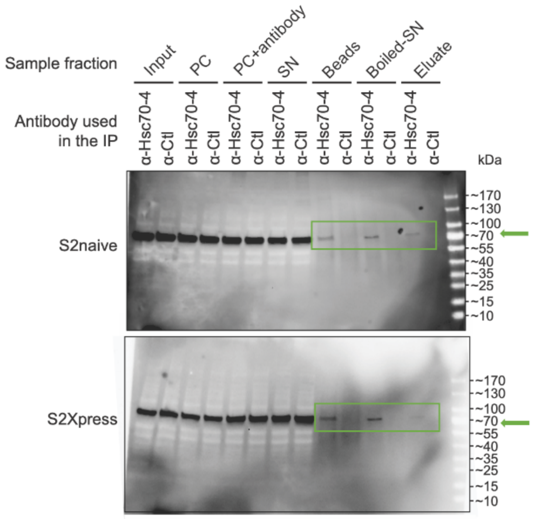

Fig. S3. High content screen intensity and silencing confirmation. (**A**) Histograms show mean + SD of median Cy3 intensity relative to the control condition (dsCtl) of the screening performed in Figure 2A. Ctl- values were subtracted from other the conditions, and values are shown relative to dsCtl. A one-way ANOVA was used to detect significant differences compared to the control condition (dsCtl) (dsCtl n=8; dsCandidates and Dyansore n=3, dsHmu n=2). (**B**) Table shows specific p-values of panel (**A**) from Figure 2. (**C**) Histograms show mean + SD of median Cy3 intensity relative to the control condition (dsCtl) of the experiment performed in Figure 2B. Ctl- values were subtracted from the other conditions and values are shown relative to dsCtl. Data are from 3 independent experiments (Dynasore n=9; dsCtl n=23, dsHsc70-4 n=23). Welch's ANOVAs followed by Dunnett's T3 post-hoc tests were used to detect significant differences compared to dsCtl. (**D**) To confirm silencing of Hsc70-4 by dsHsc70-4, S2Xpress cells were transfected with dsHsc70-4 or dsCtl (dsFLuc) for 72 h (triplicates per condition). Next, RNA was extracted, quantified, and cDNA was produced from equal amounts of RNA using Oligo(dT)18 primers. PCR was performed with primers flanking the dsRNA targeting region. PCR products were separated on a 1% agarose gel. Rp49 was used as a loading and PCR control. Silencing was confirmed by the decreased intensity of the bands from cells treated with dsHsc70-4. The experiment was performed twice, with similar results each time. (**E**) To confirm the specificity of the anti-Hsc70-4 antibody, an immunoprecipitation assay was conducted on S2naive and S2Xpress cell lysates using anti-Hsc70-4 (α-Hsc70-4) and control (α-Ctl) antibodies, along with Protein A bound to magnetic beads. Immunoblots revealed Hsc70-4 in various sample fractions collected during the immunoprecipitation. PC: pre-cleared; SN: supernatant. Histograms show mean + SD normalized to actin (unpaired t-test, n=9). *$p<0.05$; **$p<0.01$; ***$p<0.01$; ****$p<0.0001$.

Fig. S4.

A

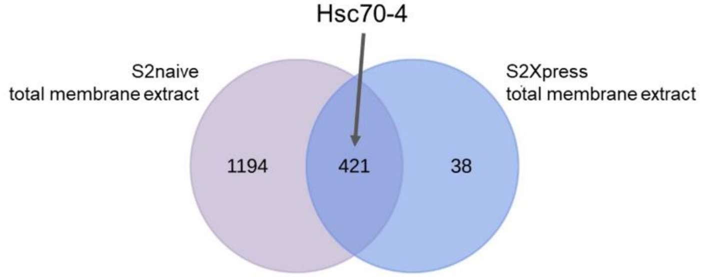

B

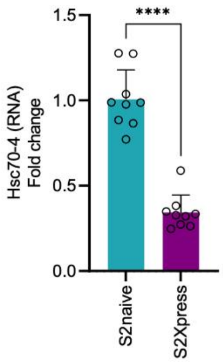

	Experiment 1			Experiment 2			Experiment 3		
	Hsc70-4	Rp49	ΔCT	Hsc70-4	Rp49	ΔCT	Hsc70-4	Rp49	ΔCT
S2naive	21,25427	18,90521	2,349063	22,75078	19,53496	3,215818	21,87553	19,03739	2,83814
S2naive	21,45901	19,02315	2,435857	22,17111	19,32201	2,849103	22,74858	19,38255	3,366028
S2naive	21,05533	18,63641	2,418926	22,99021	19,41794	3,572269	23,09307	19,69598	3,397086
S2Xpress	24,59174	20,31833	4,273413	23,47105	19,50616	3,964889	25,84137	21,01003	4,831341
S2Xpress	24,93065	20,51914	4,411514	24,80529	20,22211	4,583182	25,81078	20,69543	5,115351
S2Xpress	24,58816	20,57575	4,01241	25,62947	20,85205	4,777417	24,93771	20,22961	4,708103

C

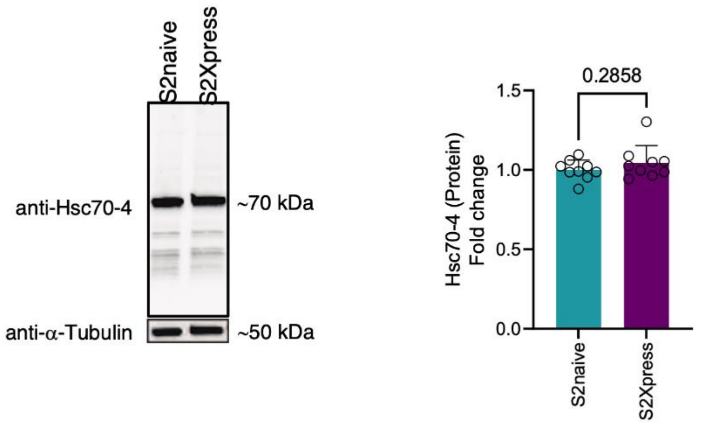

Fig. S4. Hsc70-4 expression in S2naive and S2Xpress. (**A**) Venn diagram of proteins identified in S2naive and S2Xpress total membrane extracts. Hsc70-4 was identified in both S2 cell lines. (**B**) Expression levels of Hsc70-4 between S2naive and S2Xpress cells by RT-qPCR. Rp49 was used as a housekeeping gene. Histograms show mean + SD of fold change relative to S2naive (unpaired t-test, n=9). **(C)** Immunoblot and quantification of anti-Hsc70-4 signal. Tubulin was used as loading control. Histograms show mean + SD normalized to actin (unpaired t-test, n=9). ****$p<0.0001$.

Fig. S5.

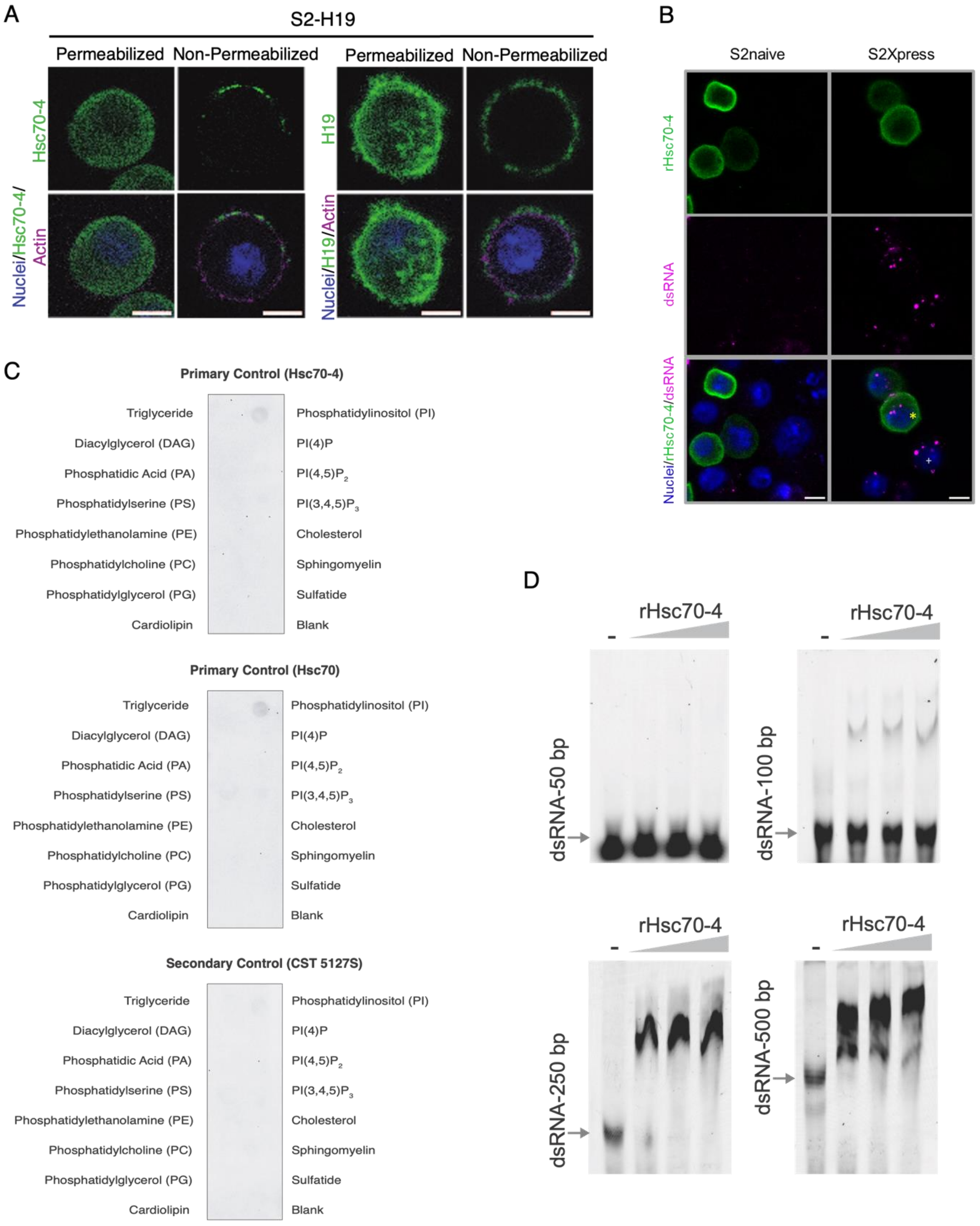

Fig. S5. Controls for immunofluorescence staining of Hsc70-4 and membrane-lipid interaction assays, and EMSA with dsRNA of different length. (**A**) Confocal

immunofluorescence images of S2-H19 cells stably expressing *C. elegans* H19 transmembrane protein. Cells were stained with anti-Hsc70-4 (left panels) and anti-H19 (right panels) under permeabilized and non-permeabilized conditions. Hsc70-4 and H19 are shown in green, actin in magenta and nuclei in blue. Images were taken as single Z-sections. (**B**) To confirm the cellular localization of recombinant Hsc70-4 overexpressed in S2 cells, S2naive and S2Xpress cells were transfected with 100 ng of pHsc70-4 for 48 h followed by soaking with 30 ng of Cy3-labeled dsRNA (dsFLuc) for 40 min. Cells were then fixed and blocked/permeabilized with 10% NGS-0.2% Triton X-100. rHsc70-4 was detected by immunofluorescence with anti-V5 (green). Nuclei are in blue. We were not able to detect a difference in dsRNA internalization between S2Xpress transfected cells overexpressing Hsc70-4 (*) and non-transfected cells (+). In S2naive cells, overexpressing Hsc70-4 did not have an effect on dsRNA internalization. Images show single Z-sections. (**C**) PIP Strips membranes incubated only with anti-Hsc70-4 primary antibody (top), only with anti-Hsc70 (human ortholog) primary antibody (middle), or secondary antibody (CST 5127S) (bottom) to control non-specific binding. (**D**) EMSAs testing the binding of dsRNA-Cy3 (dsFLuc) of different lengths (50 bp, 100 bp, 250 bp, and 500 bp) to rHsc70-4. Confocal images were taken at 630x magnification. Scale bars represent 5 µm.

Supplementary Tables

Table S1. List of proteins found in cell surface proteins purification (Fig. 1D-F).

Table S2. List of proteins found in dsRNA binding proteins immunuprecipitation (Fig. 1G, H; Fig. S2C).

Table S3. Specificity of anti-Hsc70-4. List of proteins found in the immunoprecipitation using anti-Hsc70-4 antibody (Fig. S3E).

Table S4. Proteins detected in proteomics analysis of total mebrane extracts from S2naive and S2Xpress cells (Fig. S4A).

Table S5. List of primers.
